# Heterogeneous porphyrin photosensitizers based on multiple acid–base interactions with amberlyst: high leaching stability under aqueous and non-aqueous conditions and acid-dependent spectral, solubility, and photocatalytic properties

**DOI:** 10.1039/d6ra05398j

**Published:** 2026-08-26

**Authors:** Deniz Chodari-gharehpapagh, Saeed Zakavi

**Affiliations:** a Department of Chemistry, Institute for Advanced Studies in Basic Sciences (IASBS) Zanjan 45137-66731 Iran zakavi@iasbs.ac.ir +98 24 33153232 +98 24 33153202

## Abstract

Hexaprotonated *meso*-tetra(aryl)porphyrins (aryl = 2-pyridyl, 3-pyridyl and 4-pyridyl) anchored to nanostructured Amberlyst 15, nanoAmbSO_3_H or the sodium salt (NanoAmbSO_3_Na) with an exceptionally high stability against oxidative degradation and a singlet oxygen quantum yield (*ϕ*_Δ_) of almost unity are reported as new porphyrin photosensitizers. UV-vis-NIR diffuse reflectance spectroscopy (DRS), FESEM/EDX/mapping, thermal gravimetric analysis (TGA) and nitrogen adsorption–desorption porosimetry analysis were used to characterize the nanocomposites. FESEM analysis showed an average particle size of less than 90 nm for the nanocomposites formed from the agglomeration of very small nanoparticles. The results of BET analysis were in agreement with the occupation of the smaller mesopores of the polymer by the nonplanar hexaprotonated porphyrins. The aerobic photooxidation of some environmental organic pollutants including Rhodamine B, Brilliant green and methyl phenyl sulfide was studied under aqueous conditions in the presence of the photosensitizers and their corresponding water soluble counterparts. The water-soluble hexaprotonated porphyrins showed much higher photocatalytic activity in short-term photooxidation of the organic dyes under homogeneous conditions. However, complete degradation of the porphyrins occurred in a short time interval (5 min). The polymer supported hexaprotonated porphyrins were much more efficient and stable than the non-immobilized counterparts towards oxidative degradation with singlet oxygen in both short- and long-term oxidation of the organic compounds. 1,3-Diphenylisobenzofuran as a specific chemical quencher of ^1^O_2_ (^1^Δ_g_) was used to determine the singlet oxygen quantum yield (ϕ_Δ_) of the photosensitizers. Interestingly, the hexaprotonated porphyrins immobilized on the neutralized polymer (nanoAmbSO_3_Na) showed approximately unity *ϕ*_Δ_ and higher efficiencies than those supported on nanoAmbSO_3_H (0.20–0.46).

## Abstract 2

Hexaprotonated *meso*-tetra(aryl)porphyrins anchored to nanostructured Amberlyst 15 with an exceptionally high stability against oxidative degradation and *ϕ*_Δ_ of almost unity are reported as new porphyrin photosensitizers.

## Graphical abstract

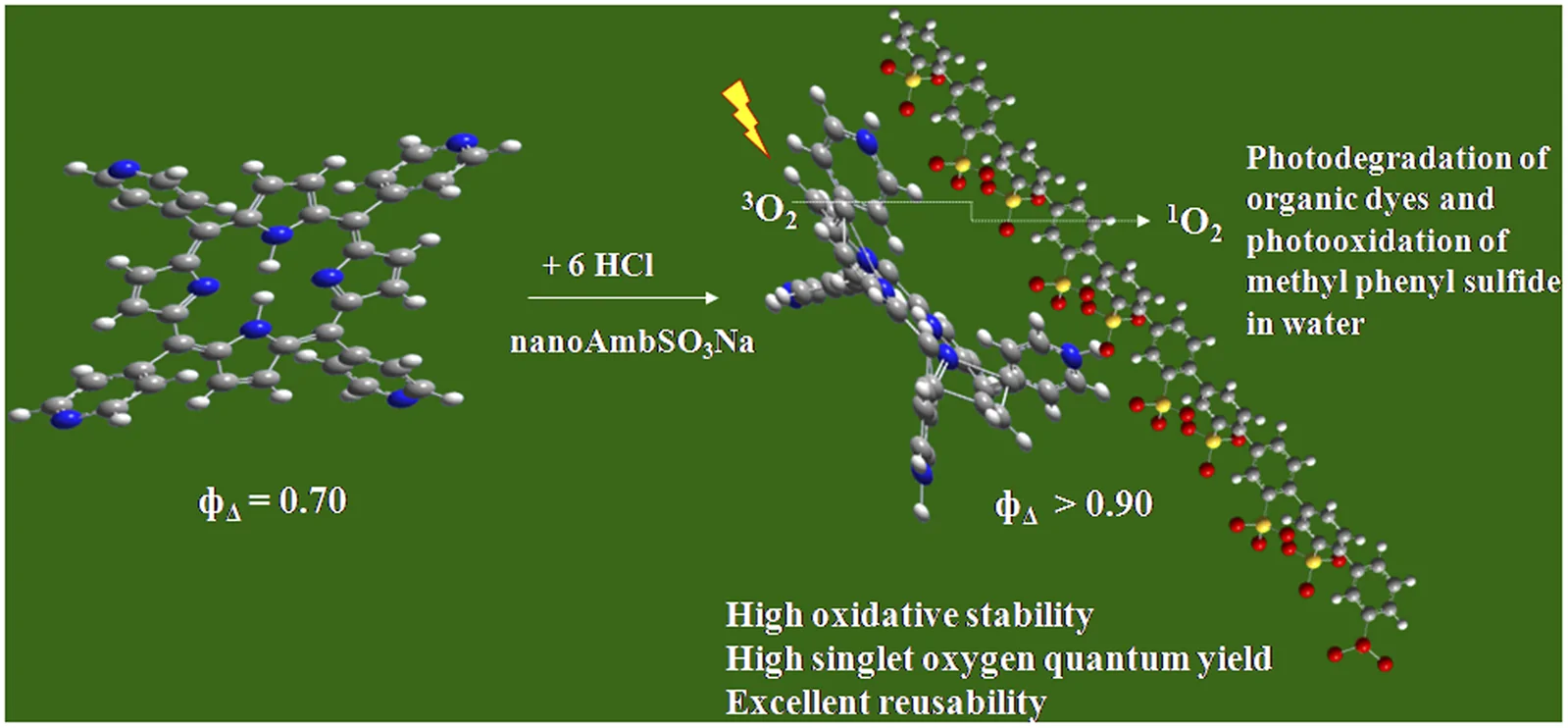

## Introduction

The ability of porphyrins for the production of reactive oxygen species (ROS) was first reported in the early 20th century.^1^ Porphyrins and porphyrinoids including phthalocyanines, chlorins and bacteriochlorins and their metal complexes have been widely used as photosensitizers in organic transformations, photodynamic therapy (PDT), antiviral photodynamic therapy, antibacterial photodynamic therapy (APDT) and dye sensitized solar cells (DSSCs).^2–6^ The very intense absorption bands of this family of aromatic macrocyclic compounds (10^−3^*ε* = 1 to 500 M^−1^ cm^−1^) appear in the visible light region.^7,8^ The excited states formed following light absorption are of appropriate energy to sensitize molecular oxygen.^9^ Furthermore, high oxidative stability towards light exposure and ROS due to the high degree of aromaticity of the porphyrin ring,^10^ proper solubility in aqueous and non-aqueous solvents depending on the peripheral groups attached to the porphyrin ring as well as tunable photophysical properties^11^ are among the most characteristic features of these photosensitizers. Porphyrins, porphyrinoids and their metal complexes are readily immobilized on the surface or into the pores of organic and inorganic supports,^12^ making it possible to exploit the advantages of heterogeneous photosensitizers. The formation of covalent bonds between the photosensitizers and solid supports such as metal organic frameworks,^13^ TiO_2_,^14^ and platinum^15^ is involved in the immobilization process. However, porphyrin photosensitizers with cationic or anionic groups at the periphery may be immobilized on ion-exchange polymers through electrostatic interactions.^16–18^ On the other hand, acid-base interactions between acidic polymers and porphyrins (or phthalocyanines^19^) have been used for the synthesis of new hybrid photosensitizers.^20^ It is noteworthy that the two pyrrolenine nitrogen atoms of porphyrins bearing lone pairs of electrons (p*K*_b_ = 9) can be protonated easily with acids such as trifluoroacetic acid.^21^ As revealed in several studies,^22,23^ hydrogen bond interactions between the porphyrin diacid and the counteranion of the respective acid play crucial roles in the stability of the diprotonated porphyrins against dissociation into the components. In the case of porphyrins bearing more than two proton accepting sites at the periphery such as *meso*-tetra(pyridyl)porphyrins, *meso*-tetra(*p*-aminophenyl)porphyrin and *meso*-(*p*-(dimethylamino)phenyl)porphyrins, the peripheral basic sites as well as the central nitrogens of the porphyrins participate in acid-base reactions to form di, tri, tetra, penta and hexaprotonated species under acidic conditions.^24–27^ The UV-vis spectral changes caused by the core and/or peripheral protonation of porphyrins allow the design of new porphyrin photosensitizers with modified absorption spectra and photophysical properties;^17,28–30^ the Soret band of porphyrins generally shows a red shift upon formation of the protonated species.^31,32^ Also, with the exception of electron-deficient porphyrins, red shifts of the *Q* bands have been observed.^33^ It is worth noting that regardless of the direction of the shift of the *Q* band, the formation of porphyrin diacids has been associated with a significant increase in the molar absorptivity of the longer wavelength bands,^34,35^ making them more suitable photosensitizers for the longer-wavelength region of the visible light. Porphyrin photosensitizers with the ability of light absorption at the longer wavelengths of the therapeutic window are of great interest in PDT due to the deeper penetration of red light into tissues.^36^

**Fig. 1:**
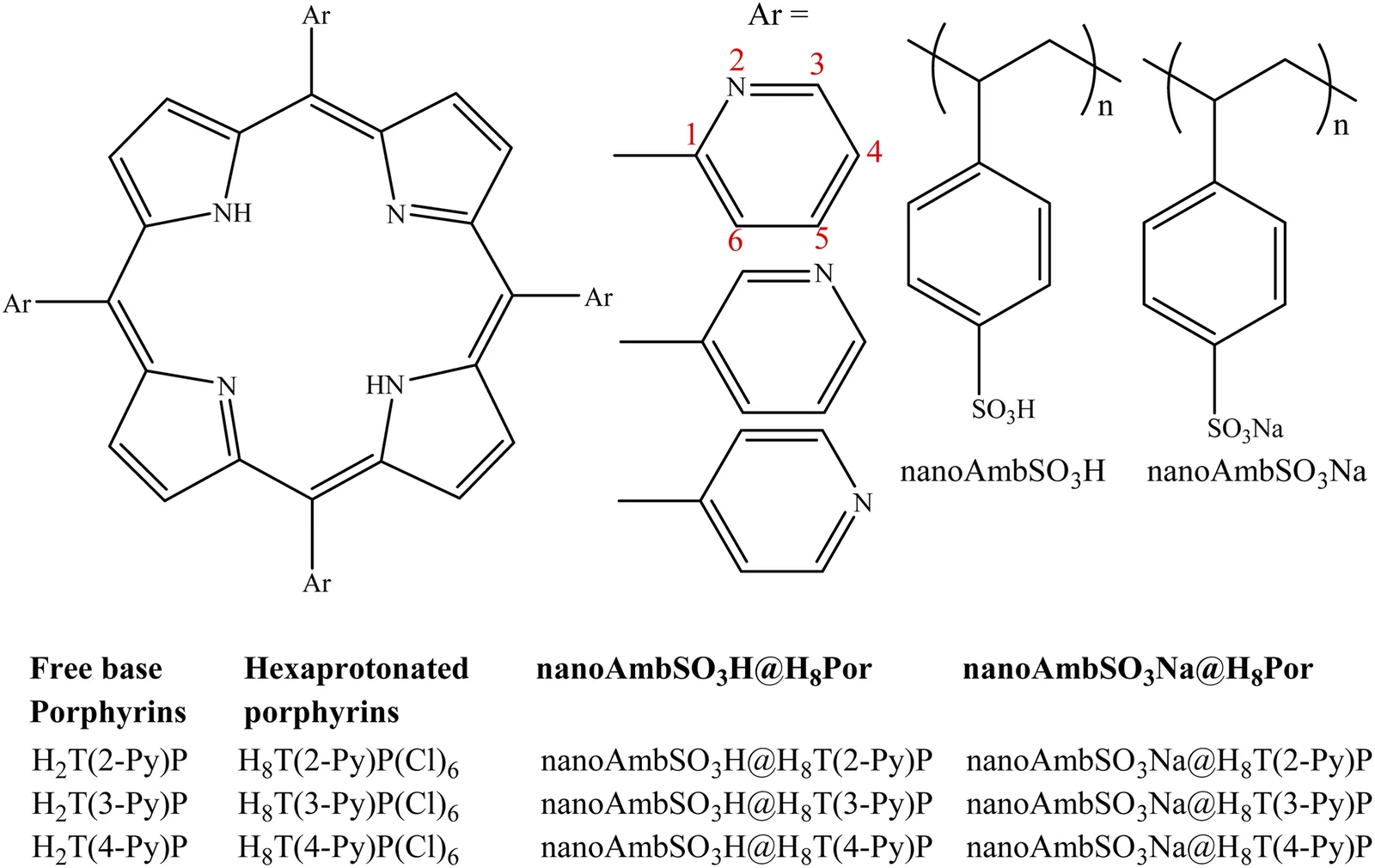
Homogeneous and heterogeneous photosensitizers based on *meso*-tetra(pyridyl)porphyrins and nanostructured Amberlyst 15 used in this study.

In the present study, the hexaprotonated derivatives of *meso*-tetra(aryl)porphyrins (aryl = 2-, 3- and 4-pyridyl) anchored to a porous nanostructured cation (proton or sodium) exchange polymer, *i.e.,* Amberlyst 15 have been used as new porphyrin photosensitizers for aerobic oxidation of organic dyes pollutants^37–39^ including Rhodamine B (RhB) and Brilliant green (BG) (Fig. 1). Also, the oxidation of a representative organic sulfide (methyl phenyl sulfide) was studied.

The nanostructured polymer (nanoAmbSO_3_H and the sodium salt, nanoAmbSO_3_Na) was synthesized using a top-down method and reported in 2018.^40^ Also, the nanostructured polymer was found to have a porous structure with a mean pore diameter of 24 to 30 nm. Due to the basicity of the porphyrin core and pyridyl substituents of *meso*-tetra(pyridyl)porphyrins leading to the formation of the hexaprotonated derivatives of these porphyrins under strong acidic conditions,^24^ shaking of *meso*-tetra(pyridyl)porphyrins with the strong solid-acid^41^ led to the formation of hexaprotonated porphyrins with no detectable leaching of the porphyrins from the solid support after long-term exposure of the nanocomposites to water and most organic solvents under dark or light exposure. Accordingly, the nanostructured mesoporous polymer was used for the immobilization of the porphyrins bearing six basic sites. Furthermore, the hybrid compounds have the capability of absorbing light in a wider range of the visible region than the corresponding non-immobilized free base porphyrins. On the basis of the literature,^42–46^ the oxidation and photooxidation of malodorous sulfides present in wastewater facilitate the separation of the products by adsorption techniques due to the greater polarity or polarizability of the products than that of the corresponding sulfides.

The main difference between the present amberlyst/porphyrin nanocomposites and previous ones is multiple acid–base interactions with Amberlyst leading to high leaching stability under aqueous and non-aqueous condition. In most previous studies, the *N*-methylated derivatives of *meso*-tetra(pyridyl)porphyrins were immobilized on cation exchange supports.^16,20^ The synthesis of methylated porphyrins requires more complicated synthetic procedures and purification steps. However, the immobilization of *meso*-tetra(pyridyl)porphyrins on the acidic polymer is straightforward one-step reaction which meets the requirements of green chemistry. Also, the formation of porphyrin hexaprotonated derivatives leads to large shifts of the Soret band to the longer wavelengths of the visible light and significant increase in the intensity of the longer-wavelength absorption bands of the porphyrins. The latter spectral shifts make the nanocomposites more efficient photosensitizers in deeper water and wastewater treatment processes, due to the better penetration of the longer wavelength light^36^ into water.

One of the problems encountered upon the oxidation of organic dyes under heterogeneous conditions is the adsorption of the dyes on the surface of the organic or inorganic support used for the immobilization of the catalyst or photocatalyst. While adsorption of organic substrates on the solid supports is of great importance in the removal of organic pollutants, extensive adsorption of dyes on the surface of the photocatalyst can interfere with the energy transfer and electron-transfer pathways involved in the activation of molecular oxygen in the presence of different photosensitizers leading to complications in comparison of the photocatalytic activity of different photosensitizers; in the present study, the nanocomposites were prepared either by direct reaction of *meso*-tetra(pyridyl)porphyrins and the acidic Amberlyst (nanoAmbSO_3_H) or through the reaction of as-prepared porphyrin hexaprotonated derivatives with the sodium salt of the polymer (nanoAmbSO_3_Na). In the case of the former, due to the low porphyrin loading used for the preparation of the hybrid compounds, several –SO_3_H groups remain on the surface of the polymer support which can contribute to the adsorption of organic substrates by hydrogen-bond formation. In the case of the latter, there are anionic sites on the neutralized polymer which can absorb organic dyes by electrostatic interactions. Due to the low and comparable adsorption of BG and RhB on the polymer support (in the form of nanoAmbSO_3_H or the sodium salt, nanoAmbSO_3_Na) in comparison with other common organic dyes (such as DHN and MB), BG and RhB were used in this study. Also, the anchored porphyrins showed significantly increased oxidative stabilities and singlet oxygen quantum yields compared to those of the corresponding non-immobilized porphyrins. Furthermore, due to the hexacationic character of the porphyrin species leading to strong electrostatic interactions between the porphyrins and the anionic sites of the polymer, no detectable leaching of the porphyrins from the polymer matrix was observed under aqueous and non-aqueous conditions and long-term light exposure.

## Experimental

### Instrumental

Details of the instruments and procedures used for the preparation of the ^1^H NMR, UV-vis and diffuse reflectance UV-vis spectra, FESEM/EDX/mapping analysis, thermal gravimetric analysis (TGA) and nitrogen adsorption–desorption porosimetry analysis were described in SI, S1.

### Synthesis of *meso*-tetrakis(2-, 3- and 4- pyridyl)porphyrin

*Meso*-tetra(pyridyl)porphyrins were synthesized according to the literature^47^ (see SI S2 for more details). The ^1^H NMR and UV-vis spectral data are summarized in SI, S3.

#### Synthesis of H_8_T(2-Py)P(Cl)_6_, H_8_T(3-Py)P(Cl)_6_ and H_8_T(4-Py)P(Cl)_6_

The hexaprotonated derivative of the porphyrins were synthesized by the addition of hydrochloric acid (1 M, 10 mL) to the solution of the respective porphyrin (0.015 g, 0.025 mmol) in CH_2_Cl_2_ (10 mL), using the method of Stone and Fleischer^24^ with some modifications. The mixture was magnetically stirred for 60 min. The solvent and the excess acid were slowly evaporated at room temperature to obtain the green color crystals of the hexa-protonated compounds. It is noteworthy that due to the use of large excess amounts of the acid, the hexaprotonated porphyrins were prepared with a yield of 100% in the aqueous phase and no free base porphyrin remained in the dichloromethane phase. The UV-vis and ^1^H NMR spectra of the free base porphyrins and their hexaprotonated derivatives are shown in SI Fig. S1 to S4.

H_8_T(2-Py)P(Cl)_6_: ^1^H NMR (400 MHz, D_2_O, TMS), *δ*/ppm: 8.54–8.58 (dd, *J* = 8.56 Hz, 8H_*meta*,*para*_), 9.06–9.08 (d, *J* = 9.07 Hz, 4H_*ortho*_), 9.12 (8H_β_, s), 9.28–9.30 (dd, *J* = 9.29 Hz, 4H_*meta*_). The Soret, *Q*(0,0) and *Q*(0,1) bands (in water) appeared at 440, 581 and 633 nm, respectively.

H_8_T(3-Py)P(Cl)_6_: ^1^H NMR (400 MHz, D_2_O, TMS), *δ*/ppm: 8.65–8.66 (m, *J* = 8.57 Hz, 4H_*meta*_), 9.30–9.31 (d, *J* = 9.31 Hz, 4H_*ortho*_), 9.13 (8H_β_, s), 9.49–9.51 (d, *J* = 9.50 Hz, 4H_*para*_), 9.82 (4H_*ortho*_, s, close to the pyridine nitrogen). The Soret, *Q*(0,0) and *Q*(0,1) bands appeared at 439, 582 and 635 nm, respectively.

H_8_T(4-Py)P(Cl)_6_: ^1^H NMR (400 MHz, D_2_O, TMS), *δ*/ppm: 8.96–8.98 (dd, *J* = 8.97 Hz, 8H_*ortho*_), 9.09 (8H_β_, s), 9.25–9.26 (dd, *J* = 9.26 Hz, 8H_*meta*_). The Soret, *Q*(0,0) and *Q*(0,1) bands appeared at 442, 590 and 641 nm, respectively.

It is noteworthy that due to the intermolecular hydrogen bonds between the NH protons and water molecules, the NH resonance was not observed in D_2_O.

#### Synthesis of nanoAmbSO_3_H@H_8_T(2-Py), nanoAmbSO_3_H@H_8_T(3-Py)P and nanoAmbSO_3_H@H_8_T(4-Py)P

The polymer anchored porphyrins were synthesized using two different methods. Due to the very high acidity of the –SO_3_H groups of the polymer,^48^ the porphyrins were hexa-protonated through direct reaction of the porphyrins dissolved in ethyl acetate with nanoAmbSO_3_H (0.5 g). The synthesis and characterization of the pale-yellow nanostructured polymer from the bulk counterpart was described elsewhere;^16^ overnight stirring of a suspension of nanoAmbSO_3_H and *meso*-tetra(pyridyl)porphyrins (0.025 mmol, 0.0153 g) dissolved in 25 mL ethyl acetate at room temperature led to the formation of a green solid. The solid was separated by centrifugation and washed three times with ethyl acetate. The non-immobilized porphyrin was determined by UV-vis spectroscopy using the absorbance–concentration calibration curves constructed for H_2_T(2-, 3- or 4-Py)P in the linear region of curves. A maximum loading of 0.0485 mmol per 1.0 g of the nanocomposite was determined for the porphyrins. The presence of porphyrin in the composite was confirmed by UV-vis DRS (*vide infra*).

In an alternative method, the nanoAmbSO_3_H was first neutralized with NaOH (1 M) to prepare the sodium salt of the polymer, nanoAmbSO_3_Na. The neutralized polymer was washed with distilled water to remove the unreacted NaOH. Then the hexaprotonated porphyrins prepared through the reaction of hydrochloric acid with the porphyrins were immobilized on nanoAmbSO_3_Na. Herein, the cationic porphyrins were anchored to the polymer through cation exchange with sodium. It is noteworthy that in the case of the hybrid compounds prepared by this method no acidic –SO_3_H groups remains on the polymer. No detectable difference was observed between the UV-vis DRS of the latter and that prepared using nanoAmbSO_3_H (*vide infra*).

### General oxidation procedure

Photooxidation of methyl phenyl sulfide and organic dyes (Brilliant green and Rhodamine B) was performed in a double walled glass vessel. In order to maintain a constant temperature, water was circulated through the outer jacket. Details of the reaction setup and the emission spectrum of the LED lamp were presented elsewhere.^16,17^ See SI S4 for more details. In the case of methyl phenyl sulfide, authentic samples required for the characterization of the reaction product (s) were prepared using the reported procedures.^49–51^ The results reported in this study for the oxidation of the organic substrates are the average of triplicate experiments.

### Singlet oxygen quantum yield

The singlet oxygen quantum yield of the porphyrin photosensitizers was determined chemically by aerobic oxidation of 1,3-diphenylisobenzofuran (DPBF) as a specific quencher of singlet oxygen and a 10 W red LED lamp as the light source. Details of the procedure were described elsewhere.^26,52^

## Results and discussion

### UV-vis diffuse reflectance spectroscopy

**Fig. 2:**
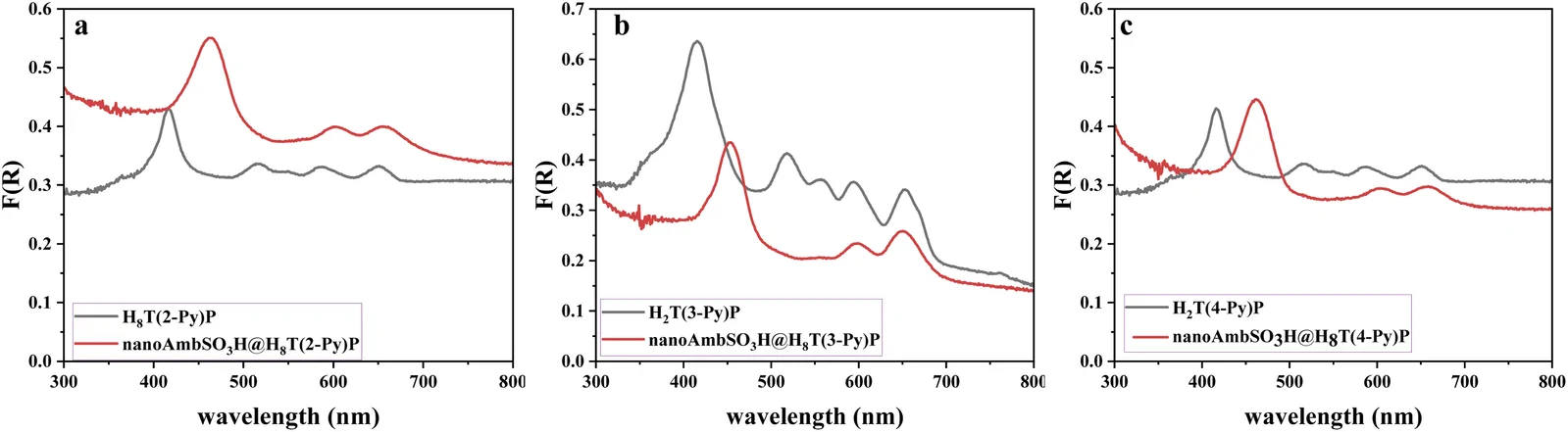
DRS of the immobilized and non-immobilized porphyrins (BaSO_4_ pellet); nanoAmbSO_3_H@H_8_T(2-Py)P (a), nanoAmbSO_3_H@H_8_T(3-Py)P (b) and nanoAmbSO_3_H@H_8_T(4-Py)P (c).

The very intense absorption bands of porphyrins in the visible region, especially in the Soret band region, allow the detection of very low concentrations of porphyrins in solutions and transparent light-transmitting composites using solution UV-vis and DRS spectroscopy. Also, the shifts in the wavelength of the Soret and Q bands of porphyrins following the formation of porphyrin derivatives such as porphyrin diacids and hexaprotonated compounds can be used to confirm the formation of these derivatives. The spectral changes upon the formation of the diprotonated and hexaprotonated derivatives of *meso*-tetra(pyridyl)porphyrins are mainly due to the out-of-plane deformation of the porphyrin core, decreased dihedral angle between the *meso*-aryl groups and porphyrin mean plane and shift of electron-density from the central and peripheral nitrogen atoms to the acid molecules which have been discussed elsewhere.^26^ As seen from the ^1^H NMR spectra (SI Fig. S2 to S4), the formation of porphyrin hexaprotonated species has been associated with the down-filed shifts of the protons of the aromatic macrocycle that is in accord with the dominance of the inductive effects due to the six HCl molecules introduced to the central and peripheral nitrogens of the porphyrins. The red shift of the Soret band of the protonated porphyrins compared to that of the free base porphyrins is in agreement with the out-of-plane deformation of the porphyrin core,^25,27^ revealed by X-ray crystallographic studies.^24^ The *Q*(0,0) band of the hexaprotonated porphyrins were significantly blue-shifted than that of the free base porphyrins, confirming a decreased electron-donation from the pyridyl substituents to the porphyrin core.^53–55^ The DRS of the free base porphyrins and their polymer supported hexaprotonated derivatives are shown in Fig. 2. Also, the solution UV-vis of the free base porphyrins and their water-soluble hexaprotonated derivatives were prepared in dichloromethane and water, respectively (SI Fig. S1). As seen from the solid state and solution spectral data summarized in Table 1, the formation of the porphyrin hexaprotonated derivative leads to a large red shift in the Soret band but red or blue shift in the *Q*(0,0) band of the porphyrins.

**Table 1:** Solid state and solution UV-vis spectral data of the free base porphyrins and their hexaprotonated species

Photosensitizer	Soret band (*λ*/nm) solution	Soret band (*λ*/nm) DRS^a^	*Q*_*x*_(1,0) band (*λ*/nm) solution	*Q*_*x*_(1,0) band (*λ*/nm) DRS^a^	*Q*_*x*_(0,0) band (*λ*/nm) solution	*Q*_*x*_(0,0) band (*λ*/nm) DRS^a^
nanoAmbSO_3_H@H_8_T(2-Py)P	—	466	—	601	—	665
nanoAmbSO_3_H@H_8_T(3-Py)P	—	463	—	591	—	652
nanoAmbSO_3_H@H_8_T(4-Py)P	—	461	—	601	—	661
H_8_T(2-Py)P(Cl)_6_^b^	440	—	581	—	633	—
H_8_T(3-Py)P(Cl)_6_^b^	439	—	582	—	635	—
H_8_T(4-Py)P(Cl)_6_^b^	442	—	590	—	641	—
H_2_T(2-Py)P^c^	416	417	588	588	647	661
H_2_T(3-Py)P^c^	418	418	590	594	646	654
H_2_T(4-Py)P^c^	420	419	590	585	665	652

a BaSO_4_ pellet.

b For the hexaprotonated porphyrins in water.

c In dichloromethane.

For a better comparison, the solid-state spectra of the free base porphyrins were also prepared in BaSO_4_ (Fig. 2). The spectra shown in Fig. 2 are in agreement with the presence of porphyrin hexaprotonated derivatives of the porphyrins in the nanocomposites. As shown in several previous studies, in addition to the large red shift of the Soret band, protonation of porphyrins decreases the number of *Q* bands to one or two bands; a relatively strong band, *Q*(0,0), is observed at longer wavelengths and a weaker band, *Q*(0,1), appears as a shoulder at the shorter wavelength side of the *Q*(0,0) band.^56–59^

### Thermal gravimetric analysis (TGA)

**Fig. 3:**
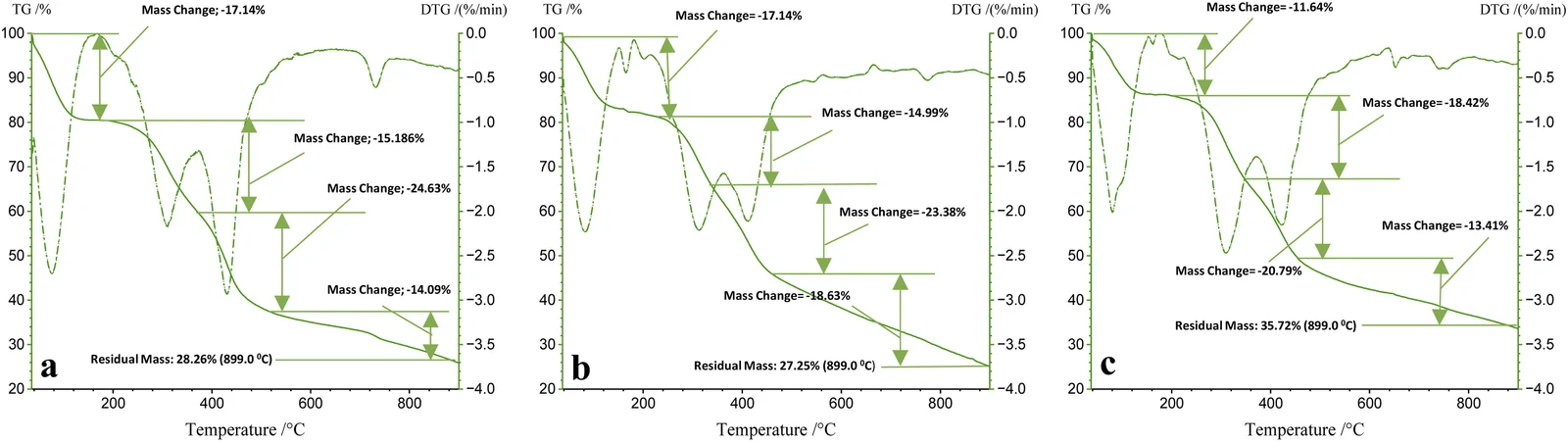
TGA curves of nanoAmbSO_3_H@H_8_T(2-Py)P (a), nanoAmbSO_3_H@H_8_T(3-Py)P (b) and nanoAmbSO_3_H@H_8_T(4-Py)P (c).

In order to investigate the effect of porphyrin hexaprotonated species on the interchain interaction of the nanostructured polymer, thermal stability of the nanostructured polymer and the nanocomposites was studied under a neutral atmosphere (Fig. 3). The acidic (nanoAmbSO_3_H) and neutralized (nanoAmbSO_3_Na) forms of the nanostructured polymer were used for the immobilization of porphyrins. As seen from Fig. 3 and SI Fig. S5, the sodium salt of the polymer (Fig. S5b) shows a relatively higher thermal stability than nanoAmbSO_3_H; the maximum weight loss of nanoAmbSO_3_Na and nanoAmbSO_3_H occurs at 462 and 446 ͦC, respectively. The incorporation of the porphyrin macrocycle in the pores of the mesoporous polymer through electrostatic interactions is associated with the replacement of the protons of the polymer –SO_3_H groups with the macrocyclic hexaprotonated species. The presence of large porphyrin molecules in the mesopores is expected to decrease the electrostatic attractive interactions between the polymer chains, leading to a decrease in the thermal stability of the polymer. On the other hand, the hexa-cationic porphyrins may play a cross-linking role to increase the stability of the polymer due to the electrostatic attractive interactions between the –SO_3_^−^ groups of the polymer chains and the cationic sites of the hexaprotonated porphyrins. As seen from Fig. 3(c–e), in spite of the porphyrin loading as low as 0.0485 mmol.g^−1^, the formation of the nanocomposites led to a decrease in the temperature of maximum weight loss rate from *ca.* 450 to 280–320 °C. This observation has been attributed to the increased interchain distance due to the presence of large porphyrin molecules between the polymer chains.

### Porosimetry analysis

**Fig. 4:**
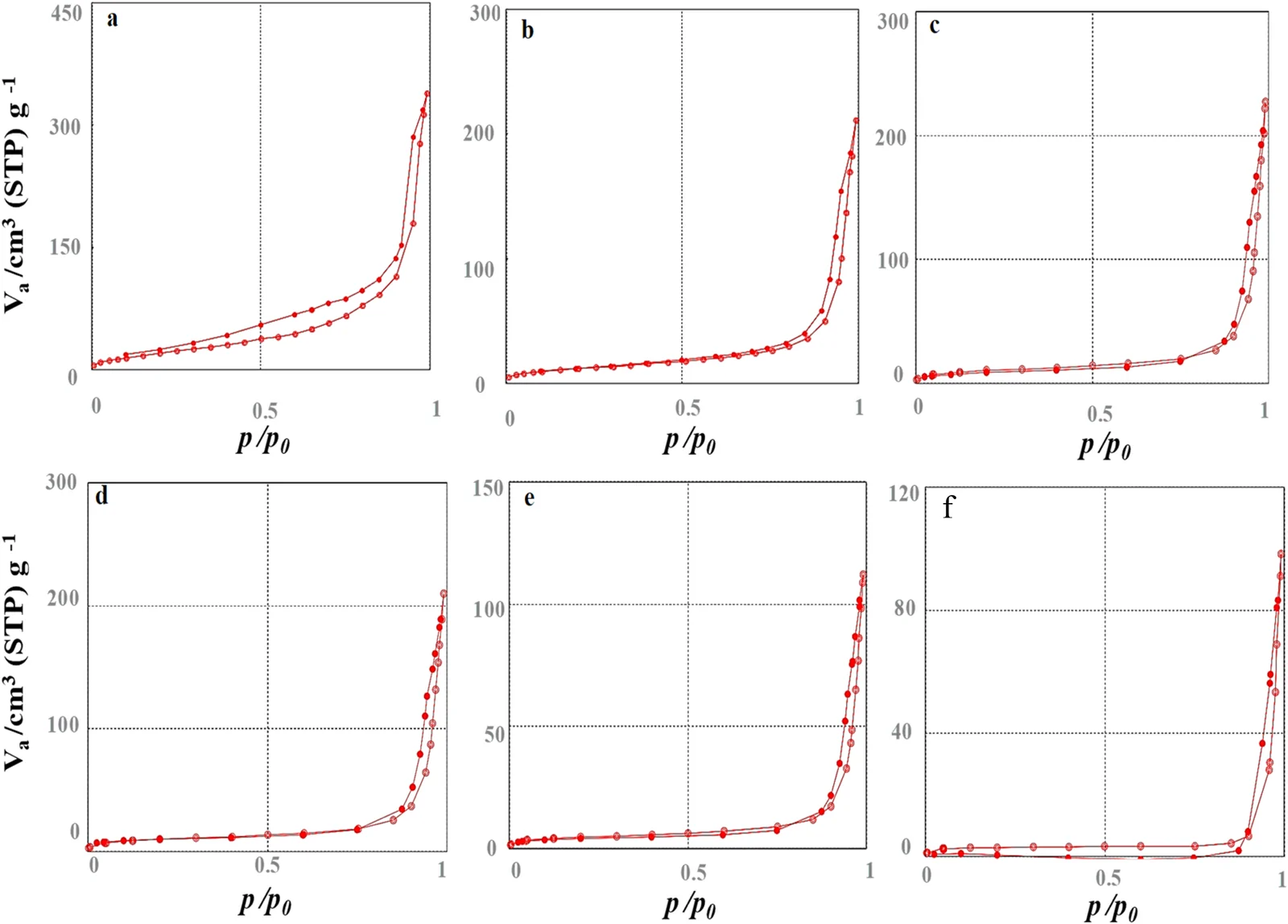
N_2_ adsorption/desorption isotherm of nanoAmbSO_3_H (a), nanoAmbSO_3_Na (b), nanoAmbSO_3_H@H_8_T(2-Py)P (c), nanoAmbSO_3_H@H_8_T(3-Py)P (d), nanoAmbSO_3_H@H_8_T(4-Py)P (e) and nanoAmbSO_3_Na@H_8_T(4-Py)P (f).

The nanostructured polymer used in this study is a mesoporous polymer with a mean pore diameter of 24.4 and 29.5 (Table 2 and Fig. 4) in the form of nanoAmbSO_3_H and nanoAmbSO_3_Na, respectively. The immobilization of the hexaprotonated porphyrins led to a simultaneous decrease in *S*_BET_ and *V*_t_ which consists with the incorporation of the porphyrins into the pores of the polymer (Table 2, entries 3–5). Also, the hybrid compounds have greater mean pore diameter which is probably due the occupation of the smaller mesopores of the polymer by the hexaprotonated porphyrins (see SI Fig. S6 for the BJH curves). The use of smaller pores leads to stronger electrostatic interactions between the cationic porphyrins and the anionic sites of the polymer and increase in the population of the larger size pores.

**Table 2:** BET analysis of the nanocomposites

Entry	Photosensitizer	*S*_BET_ a (m^2^ g^−1^)	*V*_t_ a (cm^3^ g^−1^)	Mean pore diameter (nm)
1	nanoAmbSO_3_H	85.03	0.52	24.4
2	nanoAmbSO_3_Na	42.28	0.31	29.5
3	nanoAmbSO_3_H@H_8_T(2-Py)P	36.08	0.34	38.1
4	nanoAmbSO_3_H@H_8_T(3-Py)P	34.73	0.32	36.6
5	nanoAmbSO_3_H@H_8_T(4-Py)P	31.08	0.34	43.6
6	nanoAmbSO_3_Na@H_8_T(4-Py)P	11.10	0.14	52.77

a Specific surface area and total pore volume, respectively.

### FESEM/EDX/mapping analysis

**Fig. 5:**
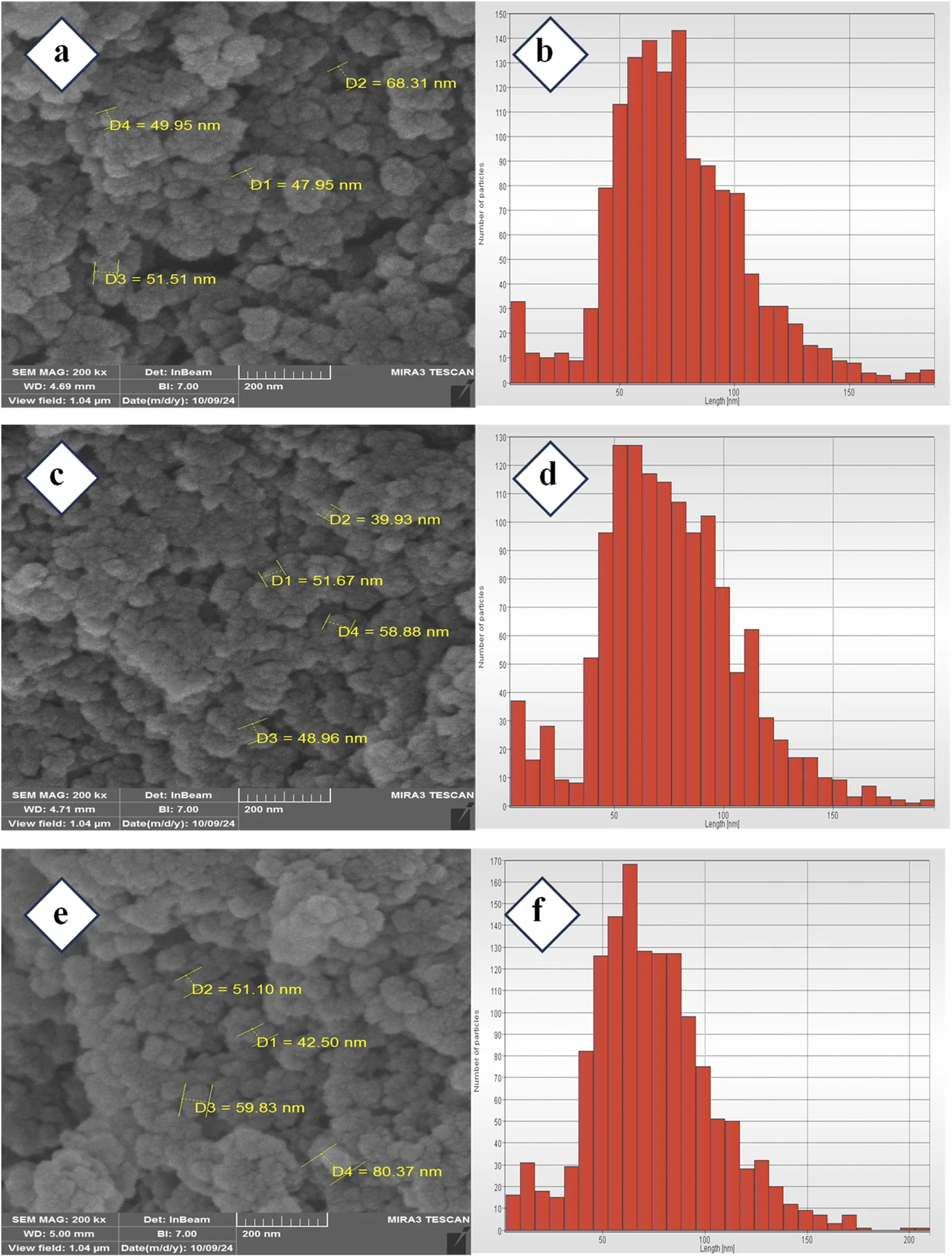
The results of FESEM analysis for the nanocomposites; nanoAmbSO_3_H@H_8_T(2-Py)P (a and b) nanoAmbSO_3_H@H_8_T(3-Py)P (c and d) and nanoAmbSO_3_H@H_8_T(4-Py)P (e and f).

**Fig. 6:**
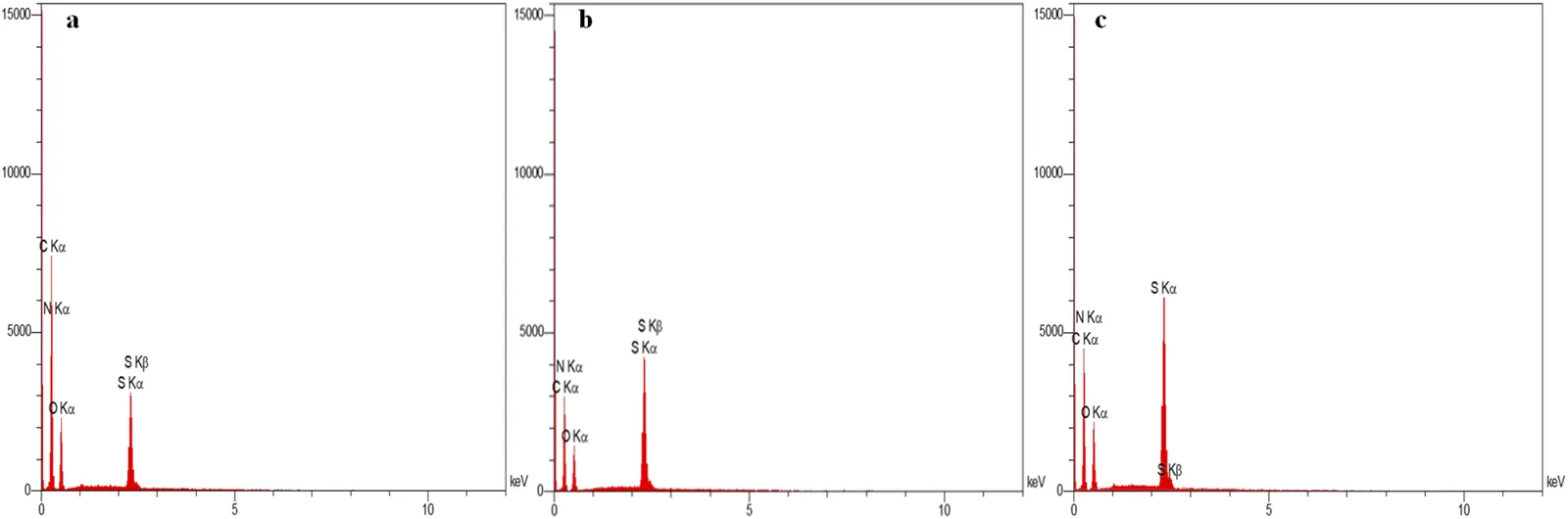
EDX spectra for (a) nanoAmbSO_3_H@H_8_T(2-Py)P, (b) nanoAmbSO_3_H@H_8_T(3-Py)P and (c) nanoAmbSO_3_H@H_8_T(4-Py)P.

**Fig. 7:**
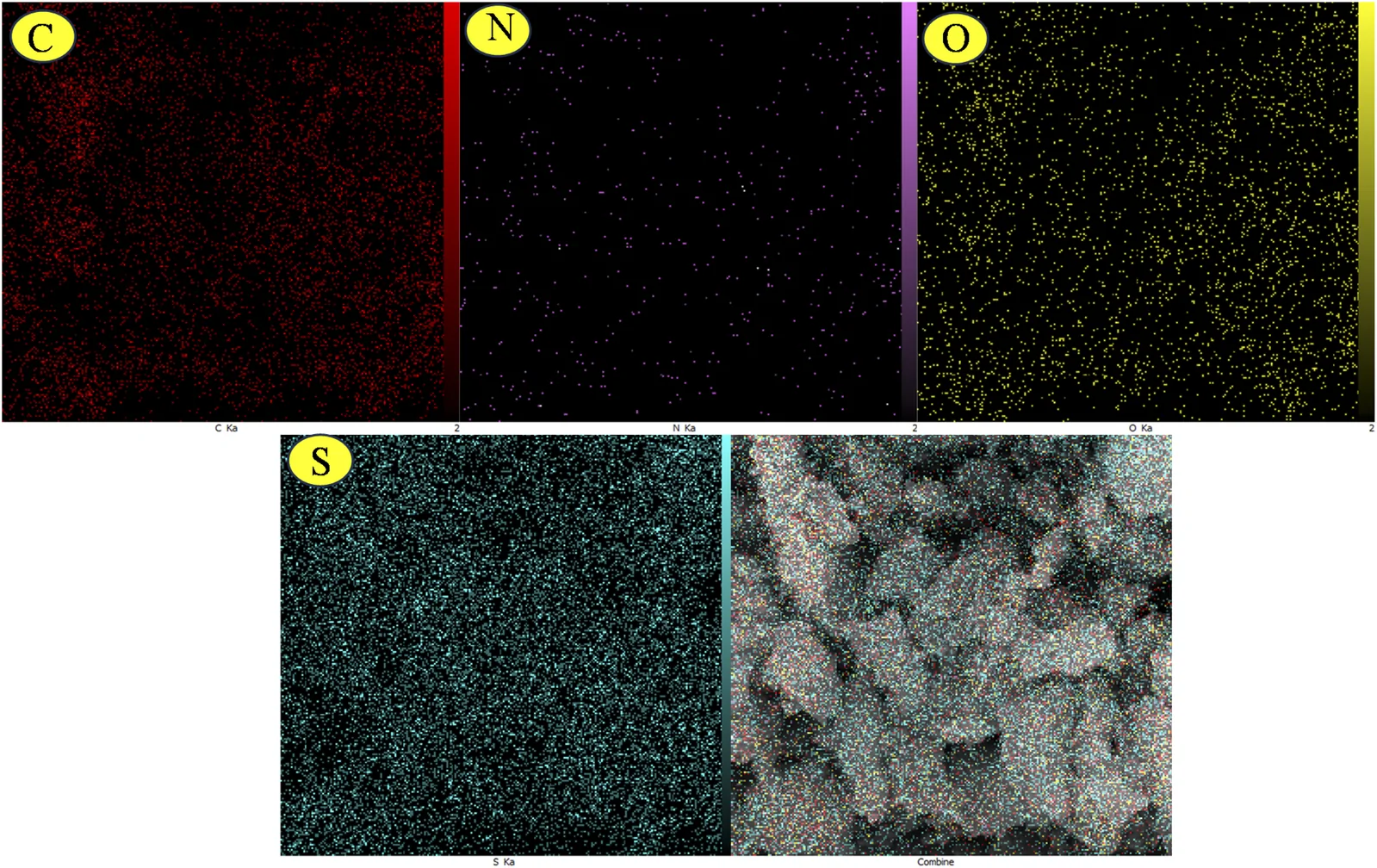
Elemental mapping of nanoAmbSO_3_H@H_8_T(2-Py)P. See SI Fig. S4 to S7 for the mapping of nanoAmbSO_3_H@H_8_T(3-Py)P and nanoAmbSO_3_H@H_8_T(4-Py)P.

The size distribution and morphology of nanoAmbSO_3_H@H_8_T(2-Py)P, nanoAmbSO_3_H@H_8_T(3-Py)P and nanoAmbSO_3_H@H_8_T(4-Py)P as well as the presence of different elements in the structure of the nanoparticles were studied by FESEM/EDX analysis (Fig. 5–7 and SI Fig. S7 to S11). The results reveal an average particle size of less than 90 nm for agglomerated nanoparticles. The UV-vis spectrum of a solution-like suspension of nanoAmbSO_3_H@H_8_T(4-Py)P in water clearly shows the Soret and *Q* bands of the porphyrin (SI Fig. S12) which is in accord with the very small particle size, evidenced from the FESEM images. The results reveal an average particle size of less than 90 nm for agglomerated nanoparticles. However, as seen from the images, the agglomerated nanoparticles consist of very small nanoparticles. As shown in SI Fig. S12, ultrasound treatment of the nanocomposites in water led to the formation of a solution-like suspension with UV-vis spectrum very similar to that of the hexaprotonated species in water. This observation is in agreement with the immobilization of the porphyrins on the acidic polymer in the form of hexaprotonated species.

### Photocatalytic activity

**Scheme 1:**
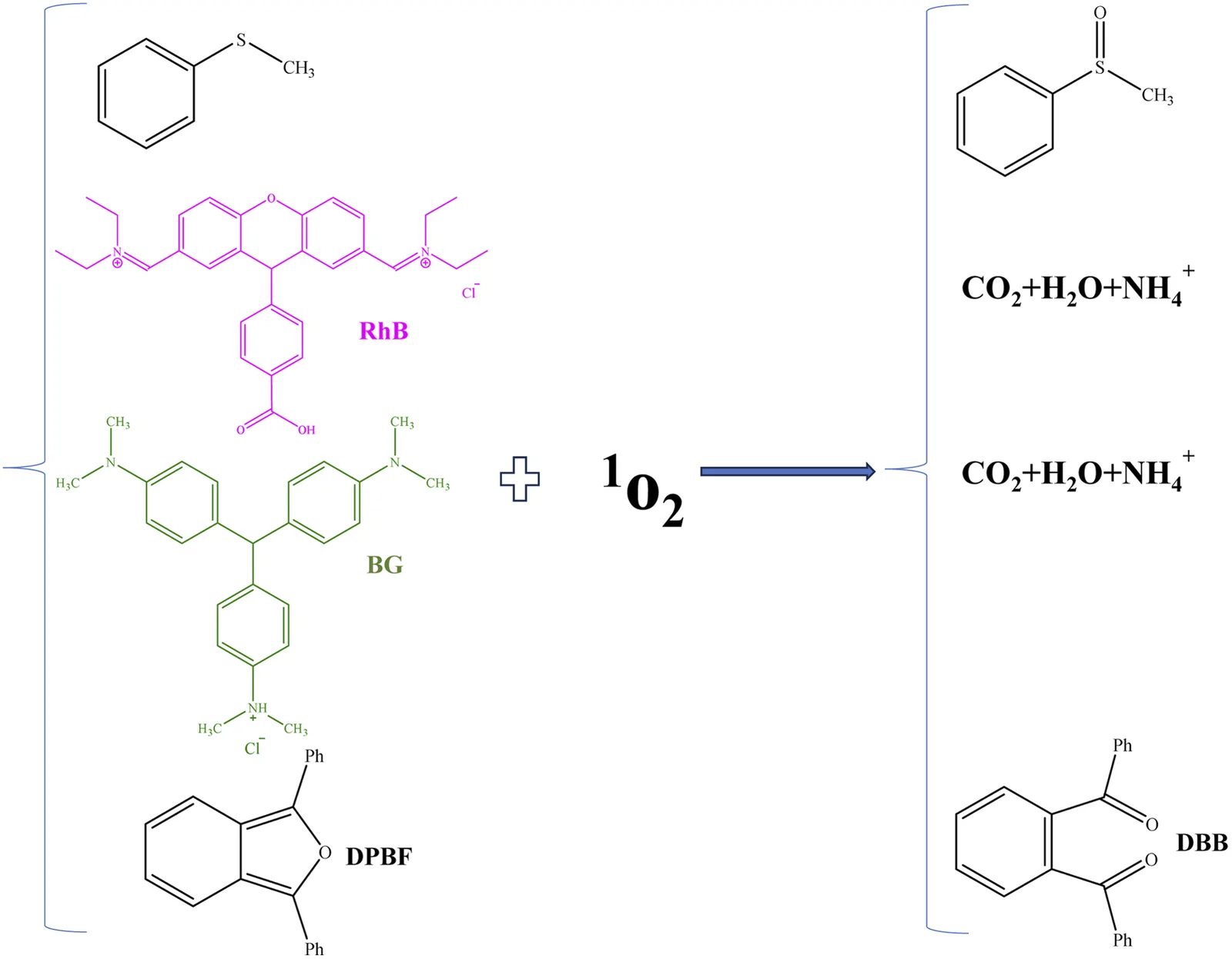
The organic compounds oxidized with singlet oxygen generated in the presence of the nanocomposites. The oxidation of DPBF to 1,2-dibenzoylbenzene (DBB) was used for the chemical determination of singlet oxygen quantum yield of the photosensitizers.

**Fig. 8:**
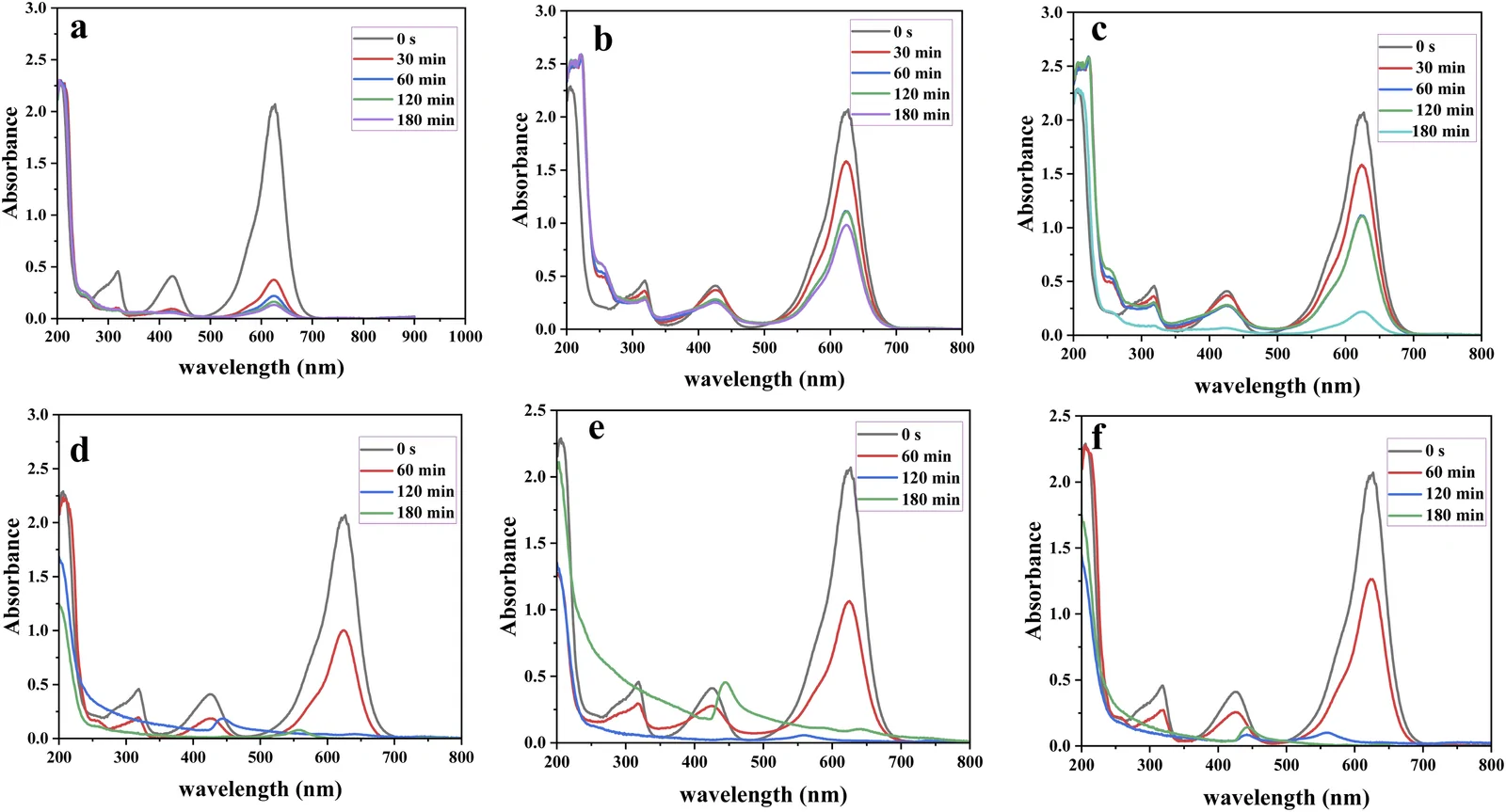
UV-vis spectral changes upon the aerobic oxidation of BG in the presence of (a) nanoAmbSO_3_H@H_8_T(2-Py)P, (b) nanoAmbSO_3_H@H_8_T(3-Py)P, (c) nanoAmbSO_3_H@H_8_T(4-Py)P (d) nanoAmbSO_3_Na@H_8_T(2-Py)P, (e) nanoAmbSO_3_Na@H_8_T(2-Py)P and (f) nanoAmbSO_3_Na@H_8_T(2-Py)P.

In order to investigate the effect of immobilization and simultaneous core and peripheral protonation of the aromatic macrocycles on their photocatalytic activity and oxidative stability, the aerobic oxidation of some organic dyes and methyl phenyl sulfide was studied (Scheme 1). The results for the oxidative degradation of BG under different reaction conditions are shown in Fig. 8, Table 3 and SI Fig. S13. Due to the insolubility of the free base porphyrins, the reactions catalyzed by these photosensitizers were performed in acetonitrile. The oxidation of the organic dye in water in the presence of the polymer supported porphyrins led to a yield of dye degradation in the range of 48 to 100%, in a reaction time of 180 min (entries 1–6).

**Table 3:** Catalytic activity and oxidative stability of the porphyrin photosensitizers in photooxidation of BG for a reaction time of 3 h in water^a^

Entry	Porphyrin	Time (min)	Pseudo-first order rate constant^b^ (10^3^ k_obs_, min^−1^)	Yield of BG degradation^c^ (%)	Catalyst degradation^d^ (%)
1	nanoAmbSO_3_H@H_8_T(2-Py)P	180	7.8	95	< 5^e^
2	nanoAmbSO_3_H@H_8_T(3-Py)P	180	5.6	48	< 5^e^
3	nanoAmbSO_3_H@H_8_T(4-Py)P	180	6.5	87	< 5^e^
4	nanoAmbSO_3_Na@H_8_T(2-Py)P	180	29.0	100	< 5^e^
5	nanoAmbSO_3_Na@H_8_T(3-Py)P	180	25.0	95	< 5^d^
6	nanoAmbSO_3_Na@H_8_T(4-Py)P	180	28.1	98	< 5^e^
7	H_8_T(2-Py)P(Cl)_6_	5	5.3	100	100
8	H_8_T(3-Py)P(Cl)_6_	5	4.8	100	94
9	H_8_T(4-Py)P(Cl)_6_	5	5.1	100	100
10	H_2_T(2-Py)P^f^	180	0.0	0	75
11	H_2_T(3-Py)P^f^	180	0.0	0	59
12	H_2_T(4-Py)P^f^	180	0.0	0	61

a For a 1 : 30 molar ratio of porphyrin to BG.

b The slope of Ln(*A*_0_/*A*_t_) *vs.* time (SI, Fig. S14).

c The average of triplicate reactions (±5%), determined on the basis of the change in the intensity of the absorption band of the dye at 625 nm (*ε* = 9928.2 M^−1^ cm^−1^); see SI, S4 for more details.

d The decrease in the intensity of the Soret band of the respective porphyrin was used to determine the catalyst degradation.

e The porphyrin released at the end of the reaction by the shaking the centrifugally separated catalyst with ammonium hydroxide solution (25%) in dimethyl sulfoxide (9 mL), was measured by UV-vis spectroscopy.

f The reactions were conducted in acetonitrile.

Interestingly, no porphyrin degradation was observed for the heterogeneous catalysts. Also, the hexaprotonated porphyrins immobilized on the sodium salt of the polymer, nanoAmbSO_3_Na (entries 4–6), were more efficient than those prepared by direct reaction of the acidic polymer, nanoAmbSO_3_H with the porphyrins (entries 1–3). Despite the very fast degradation of the dye in the presence of the water-soluble hexaprotonated porphyrins (entries 7–9) the porphyrin photosensitizers were completely degraded in 5 min. The latter hexaprotonated porphyrins were much more efficient than the corresponding free base porphyrins (entries 10–12); no photocatalytic activity was observed for the free base porphyrins in a time interval of 180 min. Furthermore, both the free base porphyrins and water-soluble hexaprotonated porphyrins were subjected to extensive degradation under the reaction conditions. It is noticeable that the oxidation of the organic dye has been shown to occur with a better efficiency under acidic conditions.^60–63^ Accordingly, the pH dependent rate of the reaction seems to be also involved in the higher yields observed in the presence of the hexaprotonated porphyrins.

### Oxidation of RhB

**Fig. 9:**
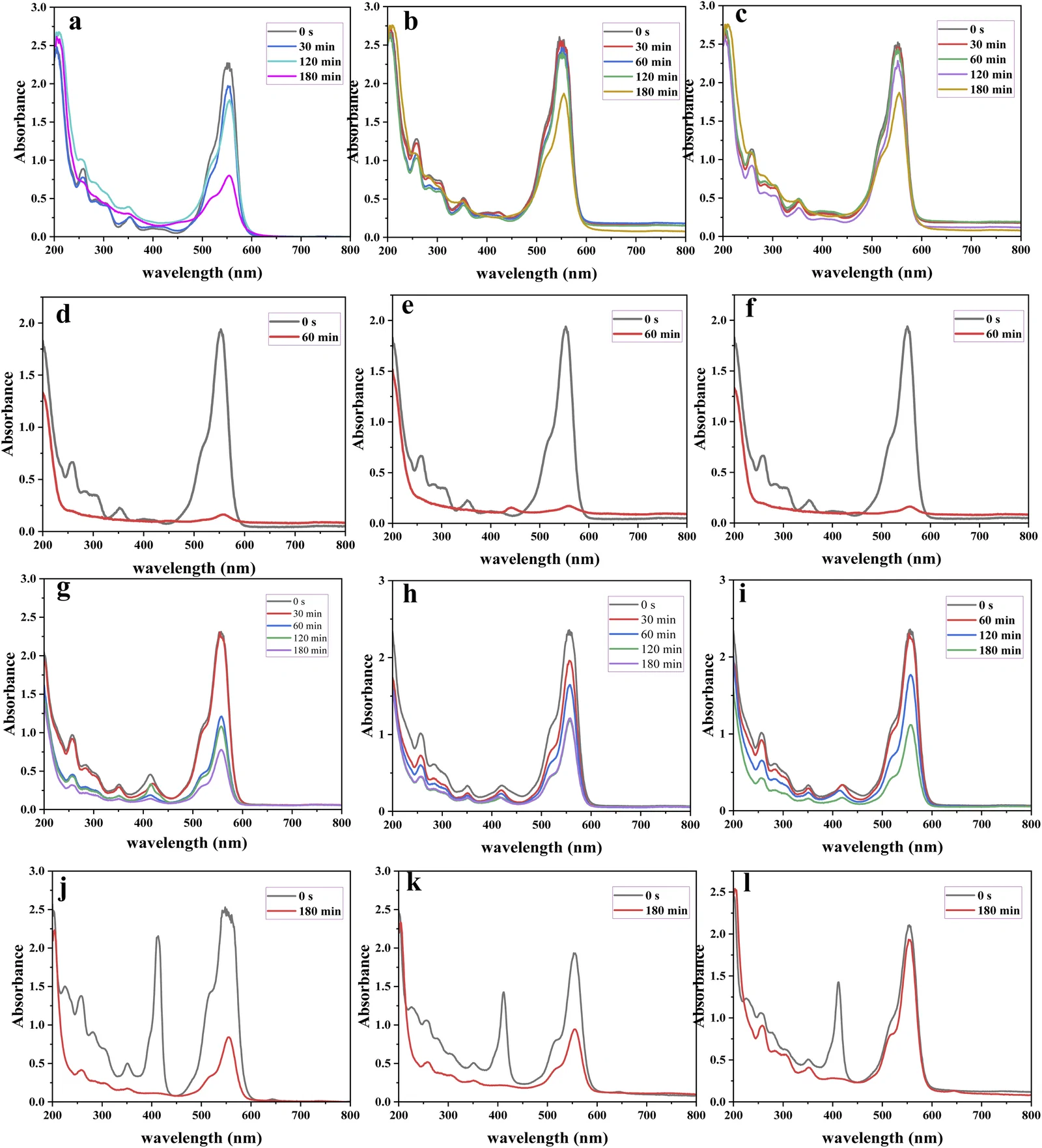
UV-vis spectral changes upon the aerobic oxidation of RhB in the presence of (a) nanoAmbSO_3_H@H_8_T(2-Py)P, (b) nanoAmbSO_3_H@H_8_T(3-Py)P, (c) nanoAmbSO_3_H@H_8_T(4-Py)P, (d) nanoAmbSO_3_Na@H_8_T(2-Py)P, (e) nanoAmbSO_3_Na@H_8_T(3-Py)P, (f) nanoAmbSO_3_Na@H_8_T(4-Py)P, (g) H_8_T(2-Py)P(Cl)_6_, (h) H_8_T(3-Py)P(Cl)_6_, (i) H_8_T(4-Py)P(Cl)_6_, (j) H_2_T(2-Py)P, (k) H_2_T(3-Py)P and (l) H_2_T(4-Py)P.

The oxidation of RhB catalyzed by the free base porphyrins and the hexaprotonated derivatives (Table 4 and Fig. 9) showed the significantly higher oxidative stability of the polymer supported hexaprotonated derivatives than that of the non-immobilized counterparts and the free base porphyrins. However, with the exception of H_2_T(4-Py)P, the free base porphyrins showed a photocatalytic activity comparable to that of the water-soluble hexaprotonated porphyrins.

**Table 4:** Aerobic oxidation of RhB in the presence of the free base porphyrins and the corresponding hexaprotonated derivatives for a reaction time of 3 h^a^

Entry	Porphyrin	Pseudo-first order rate constant^b^ (10^3^*k*_obs_, min^−1^)	Yield of RhB degradation (%)	Porphyrin degradation^c^ (%)
1	nanoAmbSO_3_H@H_8_T(2-Py)P	1.8	64	<5^c^
2	nanoAmbSO_3_H@H_8_T(3-Py)P	1.3	32	<5^c^
3	nanoAmbSO_3_H@H_8_T(4-Py)P	1.4	37	<5^c^
4	nanoAmbSO_3_Na@H_8_T(2-Py)P	31.0	98	<5^c^
5	nanoAmbSO_3_Na@H_8_T(3-Py)P	29.2	98	<5^c^
6	nanoAmbSO_3_Na@H_8_T(4-Py)P	28.3	98	<5^c^
7	H_8_T(2-Py)P(Cl)_6_	5.0	66	70
8	H_8_T(3-Py)P(Cl)_6_	3.4	43	85
9	H_8_T(4-Py)P(Cl)_6_	4.7	51	81
10	H_2_T(2-Py)P^c^	5.9	58	87
11	H_2_T(3-Py)P^c^	4.1	52	83
12	H_2_T(4-Py)P^c^	3.0	5	77

a For a 1 : 30 molar ratio of porphyrin to RhB.

b The change in the intensity of the absorption band of RhB at 553 nm (*ε* = 104 375 M^−1^ cm^−1^) was used for evaluation of the yield (The average of triplicate reactions with ± 5%); see SI, S4 for more details.

c See the caption to Table 3 and SI, Fig. S15).

Again, the heterogeneous photosensitizers prepared by the immobilization of the hexaprotonated porphyrins on the sodium salt of the polymer were more efficient than those prepared by direct immobilization of porphyrins on the acidic polymer, nanoAmbSO_3_H. The stronger electrostatic interactions between the cationic dyes (RhB and BG) and the –SO_3_^−^ groups of the neutralized polymer which brings the dye molecules in close proximity with the singlet oxygen generating sites of the polymer may be the cause of this observation. According to mechanistic studies, the oxidative degradation of RhB and BG with singlet oxygen leads to the formation of small molecules including water, carbon dioxide and ammonium.^64^

### Oxidation of methyl phenyl sulfide

The oxidation of methyl phenyl sulfide in the presence of the porphyrin photosensitizers showed an order of photocatalytic activity similar to that observed for the oxidation of the organic dyes (Table 5).

**Table 5:** Catalytic activity and oxidative stability of the porphyrin photosensitizers in photooxidation of methyl phenyl sulfide for a reaction time of 6 h^a^^,^^b^

Entry	Porphyrin	Yield^c^ (methyl phenyl sulfoxide) (%)	Catalyst degradation^b^ (%)
1	nanoAmbSO_3_H@H_8_T(2-Py)P	95	0
2	nanoAmbSO_3_H@H_8_T(3-Py)P	53	0
3	nanoAmbSO_3_H@H_8_T(4-Py)P	81	0
4	nanoAmbSO_3_Na@H_8_T(2-Py)P	99	0
5	nanoAmbSO_3_Na@H_8_T(3-Py)P	99	0
6	nanoAmbSO_3_Na@H_8_T(4-Py)P	99	0
7	H_8_T(2-Py)P(Cl)_6_	82	100
8	H_8_T(3-Py)P(Cl)_6_	60	100
9	H_8_T(4-Py)P(Cl)_6_	52	100
10	H_2_T(2-Py)P	5	100
11	H_2_T(3-Py)P	5	100
12	H_2_T(4-Py)P	5	100

a Catalyst and sulfide were used in a 1 : 3000 molar ratio.

b See the captions to Table 3.

c GC yield for triplicate reactions (±7%).; methyl phenyl sulfoxide formed as the sole product. The reactions were conducted in water (entries 1 to 9) or acetonitrile (entries 10–12).

As revealed in several studies, the singlet oxygen mediated oxidation of sulfides is significantly accelerated in the presence of hydrogen bond donors such as water, alcohols and acids. Several mechanistic studies showed that the role played by the latter additives or solvents is due to the stabilization of the persulfoxide formed as an intermediate in the mechanism of the reaction.^44,65^ The oxidation of methyl phenyl sulfide was studied in acetonitrile, due to the insolubility of the porphyrins in water. However, as shown in several previous studies,^16,17,28,66,67^ the addition of water to this solvent led to a significant increase in the yield of the reaction.

### ROS determination

**Fig. 10:**
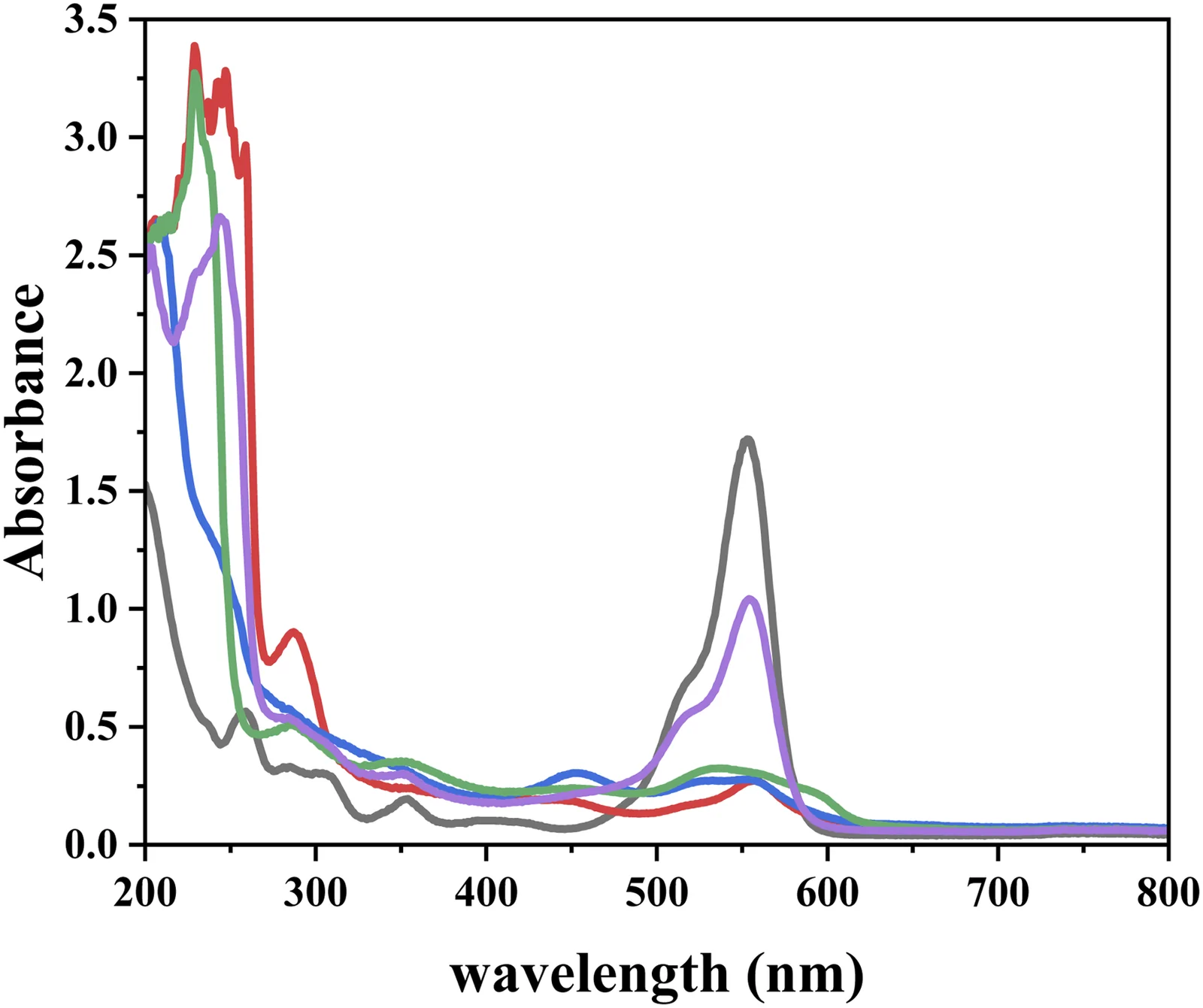
UV-vis spectral changes upon the aerobic oxidation of RhB catalyzed by nanoAmbSO_3_H@H_8_T(2-Py)P in the presence of *tert*-butanol (red curve), sodium nitrate (blue curve), potassium iodide (green curve) and 1,4-benzoquinone (pink curve), as the scavengers of hydroxyl radical, electron, hole and superoxide anion radical.

The nature of reactive oxygen species involved in the reactions was elucidated using *tert*-butyl alcohol, sodium nitrate, potassium iodide, 1,3-diphenylisobenzofuran (DPBF) and 1,4-benzoquinone, as the scavengers of hydroxyl radical, electron, hole, singlet oxygen and superoxide anion radical.^68–75^ As seen from Fig. 10, with the exception of DPBF, no decrease in the yield of the reaction was observed due to the presence of the other scavengers.

### Singlet oxygen quantum yield

**Fig. 11:**
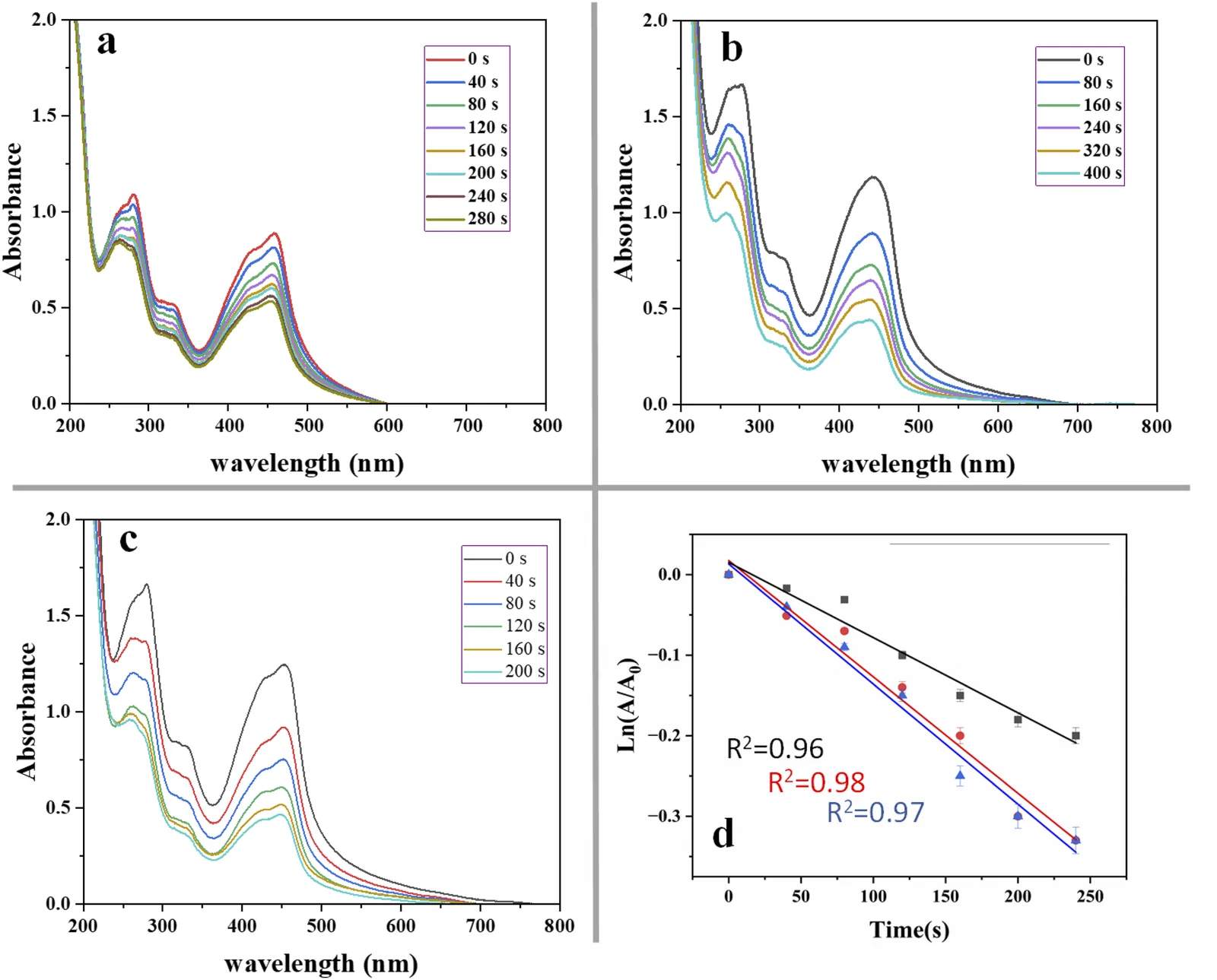
UV-vis spectral changes upon the aerobic oxidation of DPBF catalyzed by the porphyrin photosensitizers; (a) nanoAmbSO_3_H@H_8_T(2-Py)P, (b) nanoAmbSO_3_H@H_8_T(3-Py)P, (c) nanoAmbSO_3_H@H_8_T(4-Py)P and (d) the corresponding kinetics curves (grey, red and blue lines, respectively).

**Fig. 12:**
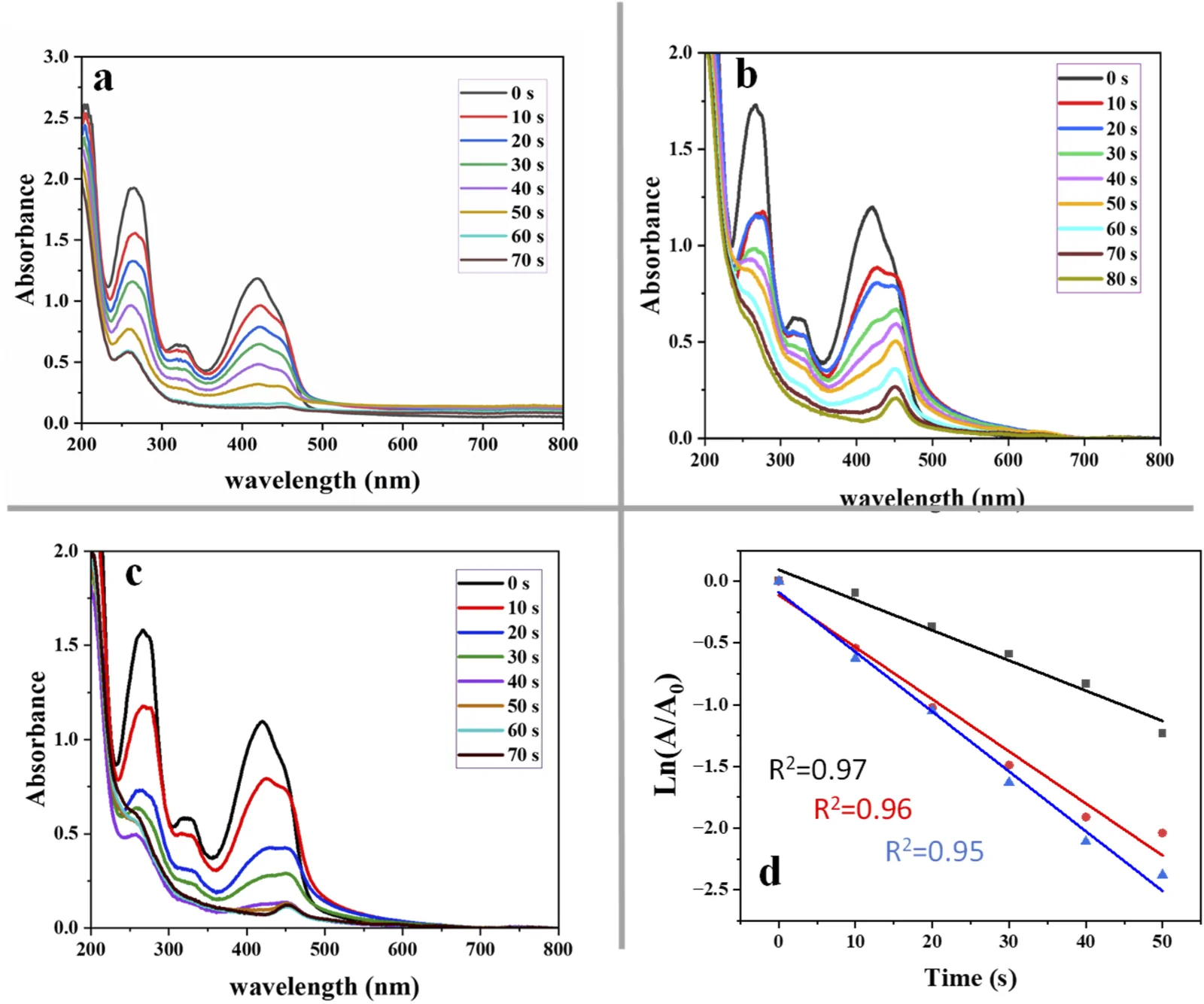
UV-vis spectral changes upon the aerobic oxidation of DPBF catalyzed by the porphyrin photosensitizers; (a) nanoAmbSO_3_Na@H_8_T(2-Py)P, (b) nanoAmbSO_3_Na@H_8_T(3-Py)P, (c) nanoAmbSO_3_Na@H_8_T(4-Py)P and the corresponding kinetics curves ((d), grey, red and blue lines, respectively).

**Fig. 13:**
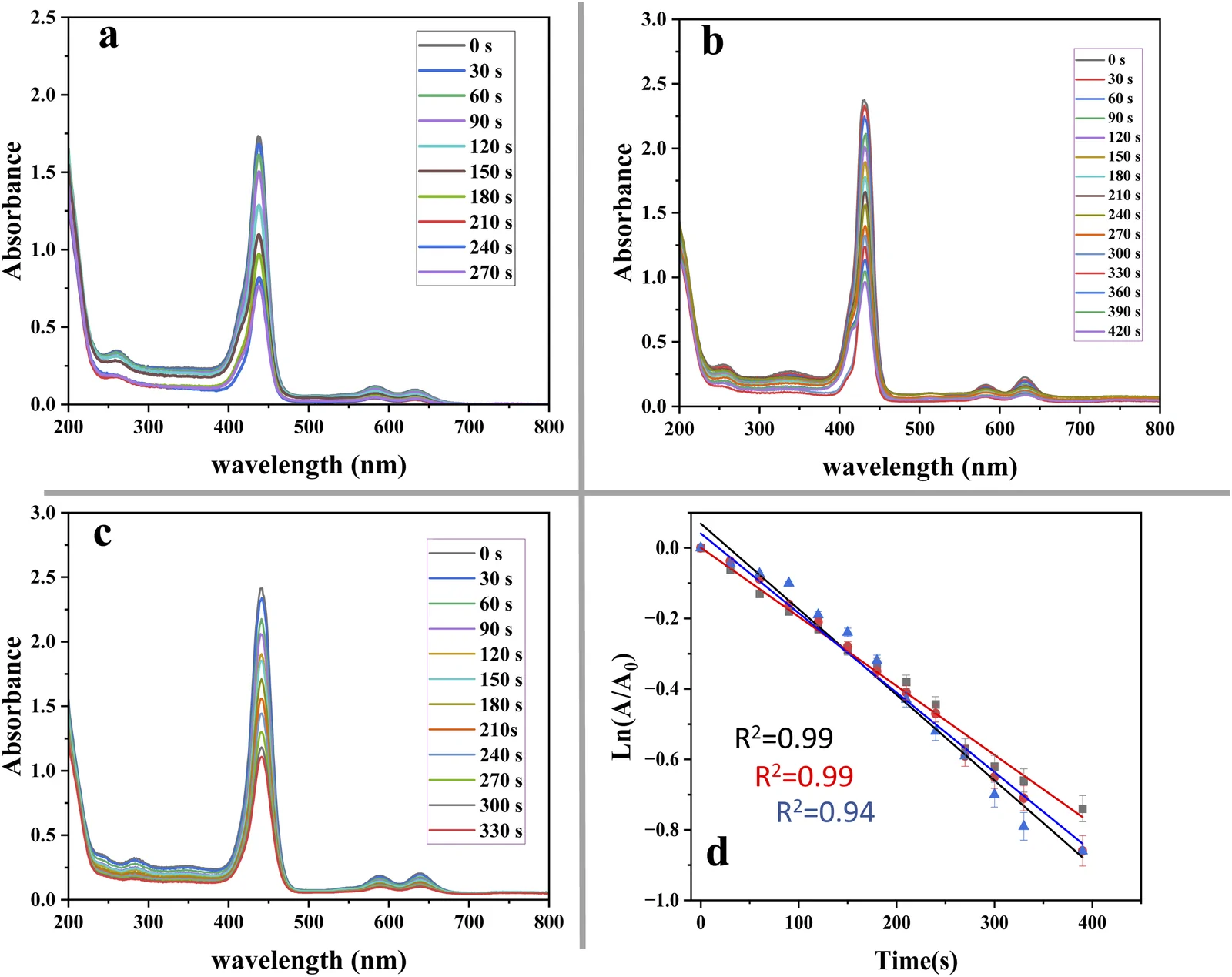
UV-vis spectral changes upon the aerobic oxidation of DPBF catalyzed by the porphyrin photosensitizers; (a) H_8_T(2-Py)P(Cl)_6_, (b) H_8_T(3-Py)P(Cl)_6_, (c) H_8_T(4-Py)P(Cl)_6_ and (d) the corresponding kinetics curves (grey, red and blue lines, respectively).

**Fig. 14:**
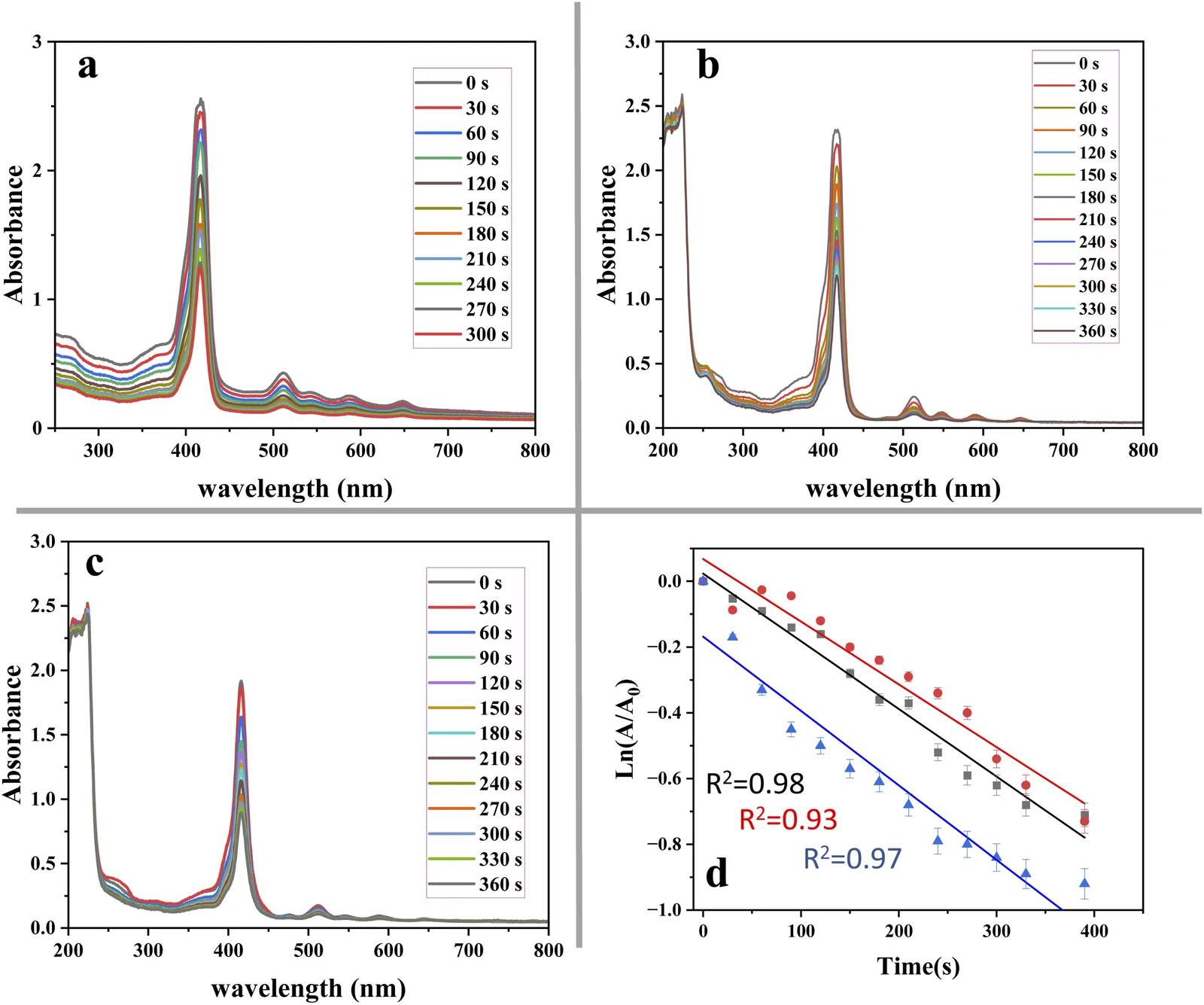
UV-vis spectral changes upon the aerobic oxidation of DPBF catalyzed by the porphyrin photosensitizers; (a) H_2_T(2-Py)P, (b) H_2_T(3-Py)P, (c) H_2_T(4-Py)P and (d) the corresponding kinetics curves (grey, red and blue lines, respectively).

The singlet oxygen quantum yield of the photosensitizers (Fig. 11–14 and Table 6) measured by the reaction of photosensitized singlet oxygen with DPBF are in agreement with the higher *ϕ*_Δ_ of the nanoAmbSO_3_Na anchored hexaprotonated porphyrins, *i.e.*, nanoAmbSO_3_Na@H_8_T(2-, 3- or 4-Py)P. The *ϕ*_Δ_ of the free base porphyrins were greater than that of the water-soluble hexaprotonated porphyrins and those supported on nanoAmbSO_3_H. As revealed from the ROS determination experiments, singlet oxygen was formed as the exclusive ROS in the presence of the porphyrin photosensitizers used in this study. Therefore, the *ϕ*_Δ_ values were determined chemically through the oxidation of DPBF^72–75^ as a specific quencher of singlet oxygen. However, due to the short reaction time (less than 7 min) used for the study of the oxidation of DPBF with singlet oxygen, no porphyrin degradation was observed. In other words, the *ϕ*_Δ_ values demonstrate the intrinsic quantum yield of the sensitizers estimated in the absence of the catalyst oxidative destruction as an unwanted side reaction. The comparison of the series of nanoAmbSO_3_Na@H_8_T(2-, 3- or 4-Py)P and nanoAmbSO_3_H@H_8_T(2-, 3- or 4-Py)P shows the negative effect of the –SO_3_H groups of nanoAmbSO_3_H on *ϕ*_Δ_; due to the low porphyrin loading, (0.0485 mmol g^−1^) most of the –SO_3_H group remain in the structure of the nanocomposites which can influence this parameter. On the other hand, the higher efficiency of nanoAmbSO_3_Na@H_8_T(2-, 3- or 4-Py)P compared to that of nanoAmbSO_3_H@H_8_T(2-, 3- or 4-Py)P correlates with the order of the *ϕ*_Δ_.

**Table 6:** Singlet oxygen quantum yields of the porphyrin photosensitizers^a^

Photosensitizer	Solvent	*k*_obs_ (s^−1^)	10^5^*v*_i_ (M s^−1^)	*ϕ*_Δ_
nanoAmbSO_3_H@H_8_T(2-Py)P	H_2_O	0.0019	0.0152	0.46
nanoAmbSO_3_H@H_8_T(3-Py)P	H_2_O	0.0015	0.0120	0.20
nanoAmbSO_3_H@H_8_T(4-Py)P	H_2_O	0.0014	0.0112	0.39
nanoAmbSO_3_Na@H_8_T(2-Py)P	H_2_O	0.0032	0.0256	>0.90
nanoAmbSO_3_Na@H_8_T(3-Py)P	H_2_O	0.0029	0.0232	>0.90
nanoAmbSO_3_Na@H_8_T(4-Py)P	H_2_O	0.0042	0.0336	>0.90
H_8_T(2-Py)P(Cl)_6_	H_2_O	0.0020	0.0160	0.46
H_8_T(3-Py)P(Cl)_6_	H_2_O	0.0022	0.0176	0.39
H_8_T(4-Py)P(Cl)_6_	H_2_O	0.0025	0.0200	0.57
H_2_T(2-Py)P	CH_3_CN	0.0022	0.0176	0.75
H_2_T(3-Py)P	CH_3_CN	0.0018	0.0144	0.64
H_2_T(4-Py)P	CH_3_CN	0.0025	0.0200	0.71
MB^b^	H_2_O	0.0044	0.0352	0.52
H_2_TPP^b^	CH_3_CN	0.0080	0.0640	0.60

a For a 1 : 10 molar ratio of porphyrin photosensitizer (8 × 10^−4^ mmol) to DPBF (8 × 10^−3^ mmol). A 10 W red LED lamp (*λ*_max_ = 615 nm) with full width at half maximum (FWHM) bandwidth of <20 nm was used as the light source.

b The standard photosensitizers.

### Photocatalyst reusability experiments

**Fig. 15:**
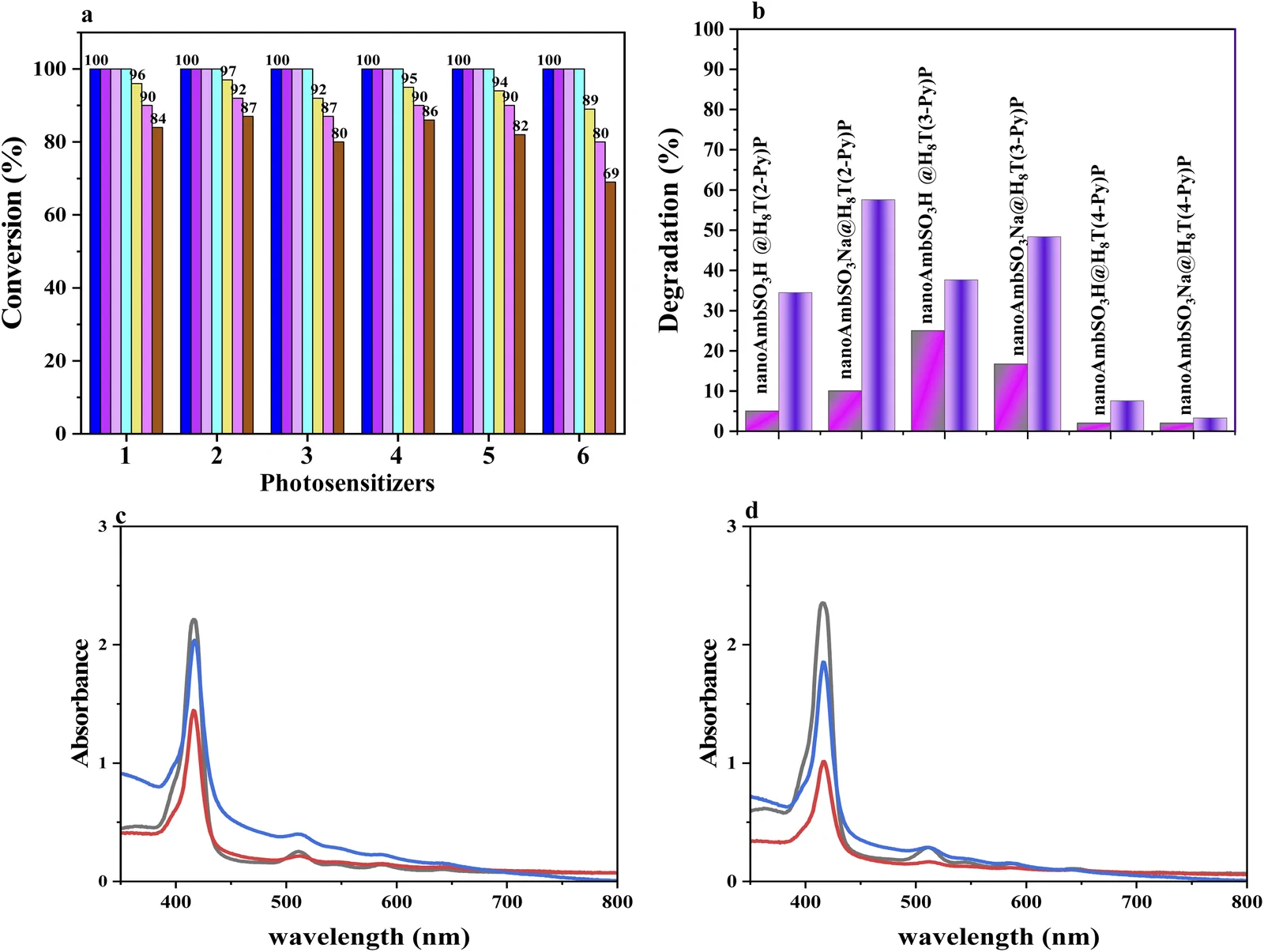
(a) The results of reusability experiments for the oxidation of methyl phenyl sulfide (MPS) in the presence of nanoAmbSO_3_H@H_8_T(2-Py)P (1), nanoAmbSO_3_Na@H_8_T(2-Py)P (2), nanoAmbSO_3_H@H_8_T(3-Py)P (3), nanoAmbSO_3_Na@H_8_T(3-Py)P (4), nanoAmbSO_3_H@H_8_T(4-Py)P (5) and nanoAmbSO_3_Na@H_8_T(4-Py)P (6). (b) The oxidative degradation of the porphyrin content of the nanocomposites, determined in the absence (blue-violet columns) and the presence (red-violet columns) of MPS. (c) Oxidative degradation of H_8_T(2-Py)P (grey curves), determined both in the absence (red curves, after 15 h) and the presence (blue curves, after 30 h) of MPS catalyzed by (c) nanoAmbSO_3_H@H_8_T(2-Py)P and (d) nanoAmbSO_3_Na@H_8_T(2-Py)P. The porphyrin was released using an ammonium hydroxide solution and the porphyrin was measured by UV-vis spectroscopy (see the caption to Table 3).

The heterogeneous catalysts showed high photooxidative stability in the oxidation of methyl phenyl sulfide. Accordingly, the reusability of the polymer supported porphyrins for the oxidation of methyl phenyl sulfide was investigated (Fig. 15a). As seen from Fig. 15a, complete oxidation of the sulfide occurred up to the 4th run. However, a decrease in the yield (12 to 30%) was observed in the 5th use of the catalysts, which seems to be mostly due to the partial loss of the catalysts in the separation step; control experiments showed a *ca.* 10–20% decrease in the catalyst weight separated at the fifth run. Also, the oxidative degradation of the porphyrins was determined. Due to the competition between the porphyrins and methyl phenyl sulfide for reaction with singlet oxygen, leading to the destruction of the porphyrins, the oxidative degradation of the porphyrins was determined both in the absence and the presence of the substrate (Fig. 15b–d and SI Fig. S16). The data summarized in Fig. 15b showed a catalyst degradation in the range of 2–25 and 3–58% for the nanoAmbSO_3_H@H_8_T(2-, 3- or 4-Py)P and nanoAmbSO_3_Na@H_8_T(2-, 3- or 4-Py)P series, respectively. As expected, the presence of the sulfide led to a remarkable decrease in the porphyrin degradation. The higher degrees of catalyst degradation in the case of the nanoAmbSO_3_Na@H_8_T(2-, 3- or 4-Py)P series are in accord with the greater *ϕ*_Δ_ of these sensitizers.

## Conclusion

The effects of simultaneous hexaprotonation and immobilization of H_2_T(2-Py)P, H_2_T(3-Py)P and H_2_T(4-Py)P into the mesopores of the acidic and sodium salt of nanostructured Amberlyst 15 on the photocatalytic activity of the porphyrins and their oxidative stability was studied and found that: (i) no leaching of the porphyrins was detected under long-term exposure of the nanocomposites to water or organic solvents; (ii) the nanocomposites were much more stable than the water-soluble hexaprotonated and the free base porphyrins towards singlet oxygen mediated oxidative degradation of the porphyrins; (iii) singlet oxygen was found to be the exclusive ROS formed in the presence of the immobilized porphyrins; (iv) the immobilization of the hexaprotonated porphyrins on the sodium salt of the polymer, nanoAmbSO_3_Na instead of nanoAmbSO_3_H led to the formation of porphyrin photosensitizers with a much higher *ϕ*_Δ_ value and higher photocatalytic activities for the immobilized porphyrins; (v) the heterogeneous porphyrin photosensitizers could be reused at least five times without loss of activity; (vi) FESEM images showed that the nanocomposites are composed of very small agglomerated nanoparticles which form solution-like suspensions in water; (vii) the increase in the mean pore diameter evidenced by BET analysis which is in contrast to the decreased specific surface area and total pore volume of the porous nanocomposites seems to be due to the accommodation of the hexaprotonated porphyrins in the smaller pores of the mesoporous polymer.

## Conflicts of interest

The authors have no conflicts to declare.

## Supplementary Material

RA-OLF-D6RA05398J-s001

## Data Availability

No other data was used for the research described in the article. The required data can be found in supplementary information (SI) file. Supplementary information: the procedures used for the synthesis and characterization of the porphyrins, the instruments and methods, general oxidation procedures used for the oxidation of the organic dyes and methyl phenyl sulfides, ^1^H NMR and UV-vis spectral data of the free base porphyrins and the hexaprotonated porphyrins with HCl, FESEM images and EDX elemental mapping of immobilized porphyrins, UV-vis spectral changes upon the aerobic oxidation of BG, UV-vis spectral changes pertaining the oxidative degradation of the porphyrins under different conditions and the UV-vis spectra of solution-like suspension of the immobilized porphyrins are presented. See DOI: https://doi.org/10.1039/d6ra05398j.
