# Supplementary material for: Heterogeneous porphyrin photosensitizers based on multiple acid–base interactions with amberlyst: high leaching stability under aqueous and non-aqueous conditions and acid-dependent spectral, solubility, and photocatalytic properties
Source: RSC Adv. 2026 Aug 26;16(42):50721–37. doi: 10.1039/d6ra05398j (PMC13511487; doi:10.1039/d6ra05398j)
Supplement: Supplementary file 1 [file RA-016-D6RA05398J-s001.pdf]

Supplementary Information (SI) for RSC Advances.
This journal is © The Royal Society of Chemistry 2026

Electronic Supplementary Information for

Heterogeneous porphyrin photosensitizers based on multiple acid-base interactions with Amberlyst: High leaching stability under aqueous and non-aqueous conditions and acid-dependent spectral, solubility, and photocatalytic properties

Deniz Chodari-gharehpapagh and Saeed Zakavi*

[a]Department of Chemistry, Institute for Advanced Studies in Basic Sciences (IASBS), Zanjan 45137-66731, Iran.

*Corresponding author. Tel.: +98 24 33153202; Fax: +98 24 33153232. E-mail address: zakavi@iasbs.ac.ir (S. Zakavi).

S1. Instrumental

S2. Synthesis of *meso*-tetrakis(2-pyridyl)porphyrin, $H_2T(2\text{-}Py)P$, *meso*-tetrakis(3-pyridyl)porphyrin, $H_2T(3\text{-}Py)P$ and *meso*-tetrakis(4-pyridyl)porphyrin, $H_2T(4\text{-}Py)P$

S3. ^1H NMR and UV-vis spectral data of the free base porphyrins.

Figure S1. UV-vis spectrum of a) $H_2T(2\text{-}Py)P$ (black curve) and $H_8T(2\text{-}Py)P(Cl)_6$ (red curve), b) $H_2T(3\text{-}Py)P$ (black curve) and $H_8T(3\text{-}Py)P(Cl)_6$ (red curve) and c) $H_2T(4\text{-}Py)P$ (black curve) and $H_8T(4\text{-}Py)P(Cl)_6$ (red curve).

S4. General oxidation procedure

Figure S2. a) ^1H NMR spectra of the aromatic region of $H_2T(3\text{-}Py)P$ (in $CDCl_3$) and $H_8T(3\text{-}Py)P(Cl)_6$ (in D_2O). The inset shows the NH region of $H_2T(3\text{-}Py)P$.

Figure S3. a) ^1H NMR spectra of the aromatic region of $H_2T(4\text{-}Py)P$ (in $CDCl_3$) and $H_8T(4\text{-}Py)P(Cl)_6$ (in D_2O). The inset shows the NH region of $H_2T(4\text{-}Py)P$.

Figure S4. a) ^{1}H NMR spectra of the aromatic region of H_2T(2-Py)P (in $CDCl_3$) and H_8T(2-Py)P$(Cl)_6$ (in D_2O). The inset shows the NH region of H_2T(2-Py)P.

Figure S5.TGA curves of (a) nanoAmbSO_3H and (b) nanoAmbSO_3Na.

Figure S6. The BJH curves for N_2 adsorption/desorption isotherm of nanoAmbSO_3H (a), nanoAmbSO_3H@H_8T(2-Py)P (b), nanoAmbSO_3H@H_8T(3-Py)P (c), nanoAmbSO_3H@H_8T(4-Py)P (d).

Figure S7. FESEM images of nanoAmbSO_3H magnified at a) 1 μm, c) 500 nm and d) 200 nm.

Figure S8. EDX elemental mapping of nanoAmbSO_3H@H_8T(3-Py)P.

Figure S9. FESEM images of nanoAmbSO_3H@H_8T(3-Py)P magnified at a) 5 μm, b) 1 μm, c) 500 nm and d,e) 200 nm.

Figure S10. EDX elemental mapping of nanoAmbSO_3H@H_8T(4-Py)P.

Figure S11. FESEM images of nanoAmbSO_3H@H_8T(4-Py)P magnified at a) 5 μm, b) 1 μm, c) 500 nm and d, e) 200 nm.

Figure S12. UV-vis spectrum of solution-like suspension of a) nanoAmbSO_3H@H_8T(2-Py)P , b) nanoAmbSO_3H@H_8T(3-Py)P , c) nanoAmbSO_3H@H_8T(4-Py)P in distilled water.

Figure S13. UV–vis spectral changes upon the aerobic oxidation of BG in the presence of a) H_2T(2-Py)P, b) H_2T(3-Py)P and c) H_2T(4-Py)P.

Figure S14. Kinetics curve for degradation of BG in the presence of the nanocomposites (a and b) and the water soluble porphyrin photosensitizers (c). No reaction was observed in the presence of the free base porphyrins in acetonitrile.

Figure S15. Kinetics curve for degradation of RhB in the presence of the nanocomposites (a and b), the water soluble porphyrin photosensitizers (c) and the free base porphyrins (in acetonitrile).

Figure S16. The oxidative degradation of the porphyrins determined both in the absence (red curve) and the presence of the substrate (blue curve), a) nanoAmbSO_3Na@H_8T(4-Py)P, b) nanoAmbSO_3H@H_8T(4-Py)P, c) nanoAmbSO_3Na@H_8T(3-Py)P and d) nanoAmbSO_3H@H_8T(3-Py)P.

S1. Instrumental

UV-vis spectroscopy

UV–vis spectra (between 200–800 nm) of the water-soluble porphyrins were prepared in water on a Pharmacia Biotech Ultraspec 4000 UV–vis spectrophotometer. The diffuse reflectance UV–vis spectra of the polymer supported porphyrins were record on a Varian Cary 5000 UV–vis–NIR absorption spectrometer was in the range of 300–800 nm.

Thermogravimetric Analysis (TGA)

Thermogravimetric analysis (TGA) was performed using a Netzsch STA 409 PC thermal analyzer under a nitrogen atmosphere. Approximately 5–10 mg of dried sample was placed in an alumina crucible and heated from room temperature to 800 °C at a heating rate of 10 °C min^{-1} under a continuous flow of [N_2 (or air)] ([~50–100] mL min^{-1}). The weight-loss behavior of the samples was recorded as a function of temperature, and the corresponding thermogravimetric curves were analyzed.

Porosimetry / N_2 adsorption–desorption (BET/BJH)

Textural properties of the samples were evaluated by N_2 adsorption–desorption measurements at 77 K using a Belsorp Max instrument (Japan). Before analysis, the samples were degassed under vacuum at 100 °C for 5 h, and the vacuum degree prior to measurement reached 3.002×10^{-6} Pa.

The specific surface area was calculated according to the Brunauer–Emmett–Teller (BET) method within the relative pressure range of P/P0=0.05–0.30. The total pore volume was estimated at P/P0 ≈ 0.99, while the pore size distribution was obtained from the desorption branch of the isotherm using the Barrett–Joyner–Halenda (BJH) method.

FESEM, EDX and Elemental Mapping

The size and morphology of the nanostructured composites were determined using a field-emission scanning electron microscope. For the preparation of the samples, a crushed portion of the nanocomposites was placed on a clean glass slide and subsequently vacuum-coated with platinum or gold to ensure conductivity and minimize charging effects during FESEM analysis. Furthermore, the digital images of the nanocomposites, along with the EDX spectra and the elemental maps of the samples, were acquired using a Hitachi S4160 field-emission scanning electron microscope operating at an accelerating voltage of 15 kV. This detailed characterization allowed for the precise evaluation of the surface topography, elemental composition, and the spatial distribution of the constituent elements within the nanostructured composites.

S2. Synthesis of *meso*-tetrakis(2-pyridyl)porphyrin, H_2T(2-Py)P, *meso*-tetrakis(3-pyridyl)porphyrin, H_2T(3-Py)P and *meso*-tetrakis(4-pyridyl)porphyrin, H_2T(4-Py)P

The porphyrins with 2- , 3- and 4-pyridyl substituents were synthesized in a yield of about 8, 13, 15%, respectively by the addition of pyrrole (2.5 mL, 36 mmol) and pyridine-2,3,4-carboxaldehyde (2.8 mL, 30 mmol) to 100 mL refluxing propionic acid. The reaction mixture was refluxed for 2 h. After cooling the mixture using an ice-bath, the solution was treated with acetone (400 mL) and 100 mL ammonia solution (aqueous, 25%), respectively. Overnight storing the mixture in freezer led to the formation of crystallized product which was collected by filtration. The solid was washed with cold MeOH and purple crystals were obtained.

S3. ^{1}H NMR and UV-vis spectral data of the free base porphyrins.

$H_2T(4\text{-}Py)P$: ^{1}H NMR (400 MHz, $CDCl_3$, TMS), δ/ppm: -2.89 (2H, br, s, NH), 8.20-8.21 (dd, J = 8.20 Hz, $8H_o$), 8.91 ($8H_\beta$, s), 9.09-9.10 (dd, J=9.11 Hz, $8H_m$). UV-vis (λ_{max}/nm) in CH_2Cl_2: 420 (Soret band), 590, 665.

$H_2T(3\text{-}Py)P$: ^{1}H NMR (400 MHz, $CDCl_3$, TMS), δ/ppm: -2.79 (2H, br, s, NH), 7.82-7.88 (dd, J = 7.82 Hz, $4H_m$), 8.58-8.61 (d, J = 8.58 Hz, $4H_o$), 8.9 ($8H_\beta$, s), 9.10-9.12 (dd, J = 9.12 Hz, $4H_p$), 9.5-9.52 ($4H_o$, s).UV-vis (λ_{max}/nm) in CH_2Cl_2: 418 (Soret band), 590, 646.

$H_2T(2\text{-}Py)P$: ^{1}H NMR (400 MHz, $CDCl_3$, TMS), δ/ppm: -2.88 (2H, br, s, NH), 7.73-7.77 (ddd, J = 7.76, Hz $4H_p$), 8.11-8.16 (td, J = 8.14, Hz $4H_m$), 8.25-8.27 (d, J = 8.26 Hz, $4H_o$), 8.91 ($8H_\beta$, s), 9.18-9.20 (ddd, J = 9.19 Hz, $4H_m$, adjacent to the pyridine nitrogen). UV-vis (λ_{max}/nm) in CH_2Cl_2: 416 (Soret band), 588, 647.

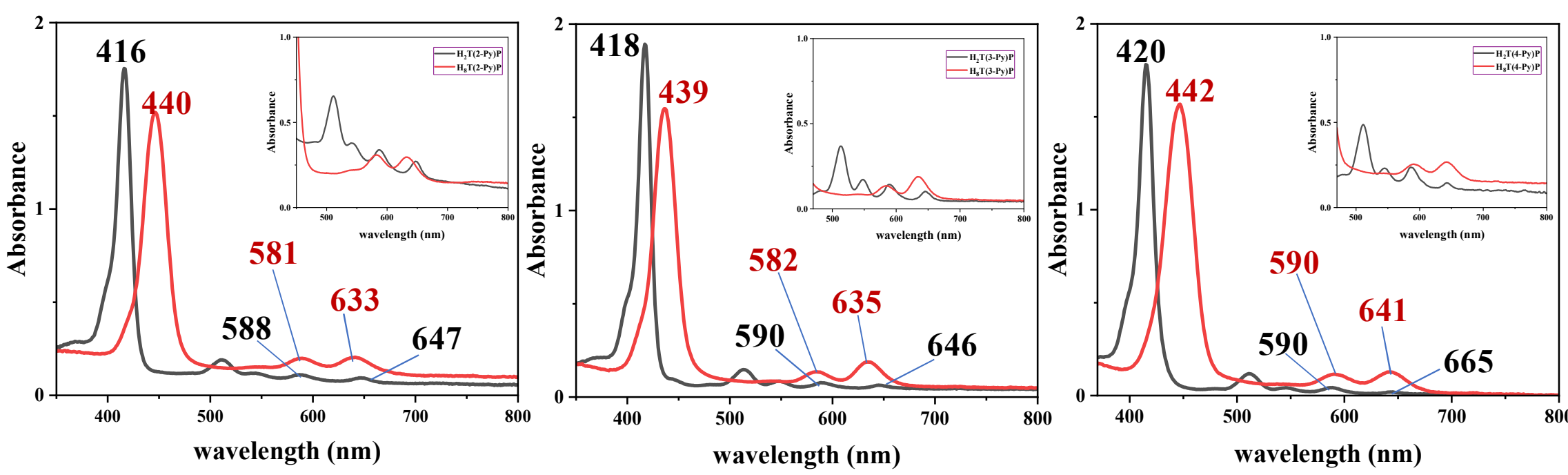

Figure S1. UV-vis spectrum of a)$H_2T(2\text{-}Py)P$ (black curve) and $H_8T(2\text{-}Py)P(Cl)_6$ (red curve), b) $H_2T(3\text{-}Py)P$ (black curve) and $H_8T(3\text{-}Py)P(Cl)_6$ (red curve) and c) $H_2T(4\text{-}Py)P$ (black curve) and $H_8T(4\text{-}Py)P(Cl)_6$ (red curve).

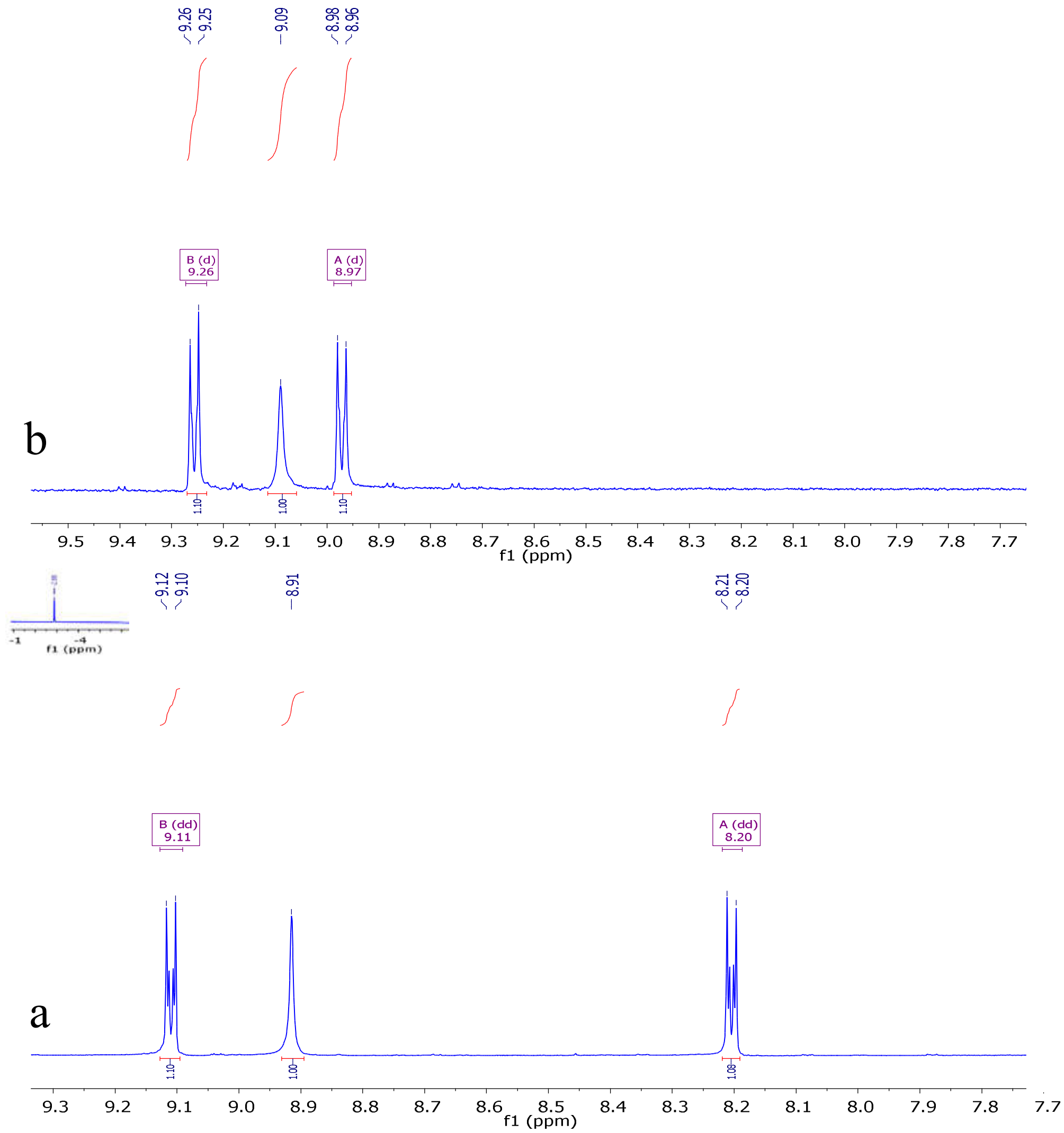

Figure S2. a) ^{1}H NMR spectra of the aromatic region of $H_2T(4\text{-}Py)P$ (in $CDCl_3$) and $H_8T(4\text{-}Py)P(Cl)_6$ (in D_2O). The inset shows the NH region of $H_2T(4\text{-}Py)P$.

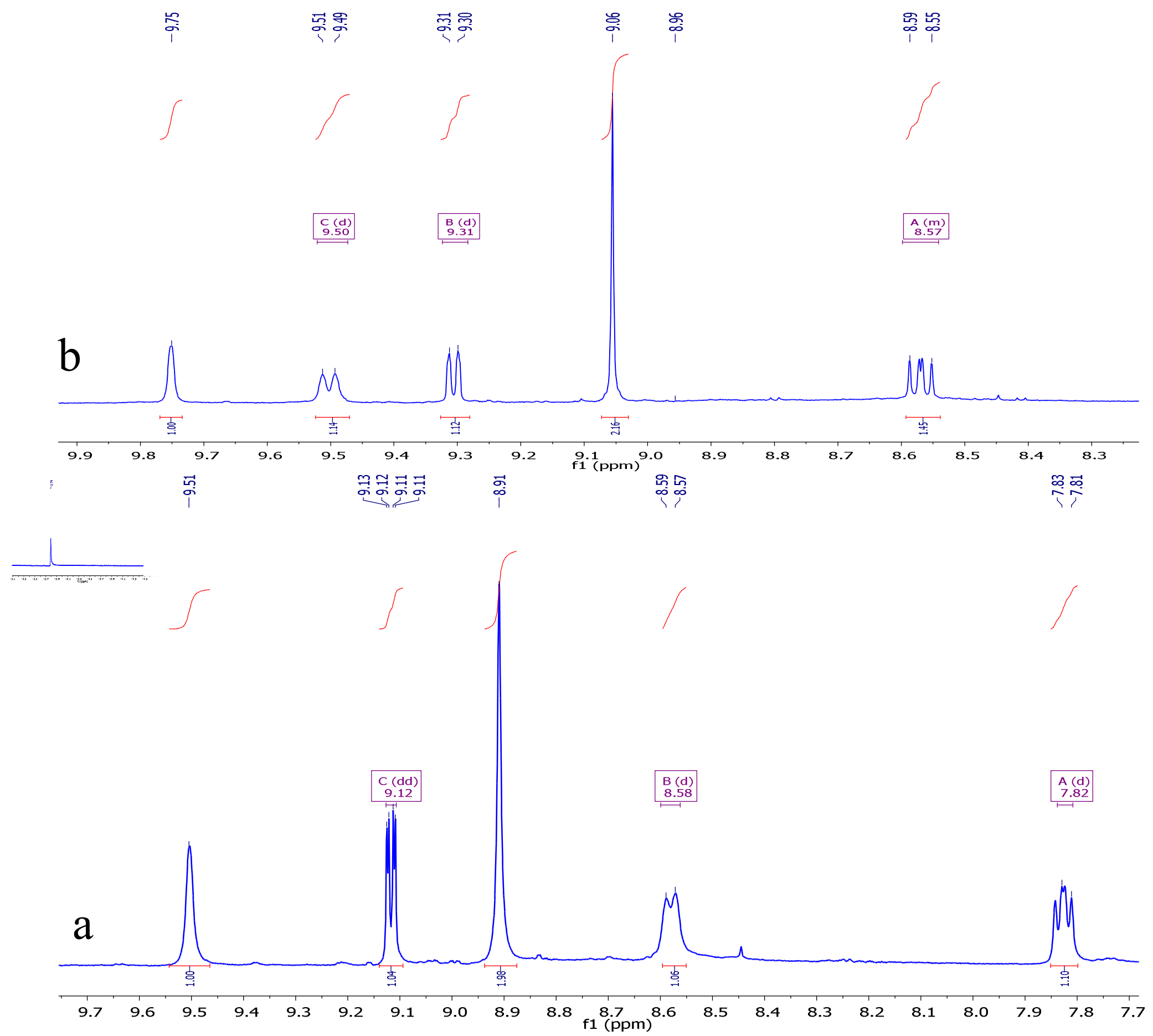

Figure S3. a) ^{1}H NMR spectra of the aromatic region of $H_2T(3\text{-}Py)P$ (in $CDCl_3$) and $H_8T(3\text{-}Py)P(Cl)_6$ (in D_2O). The inset shows the NH region of $H_2T(3\text{-}Py)P$.

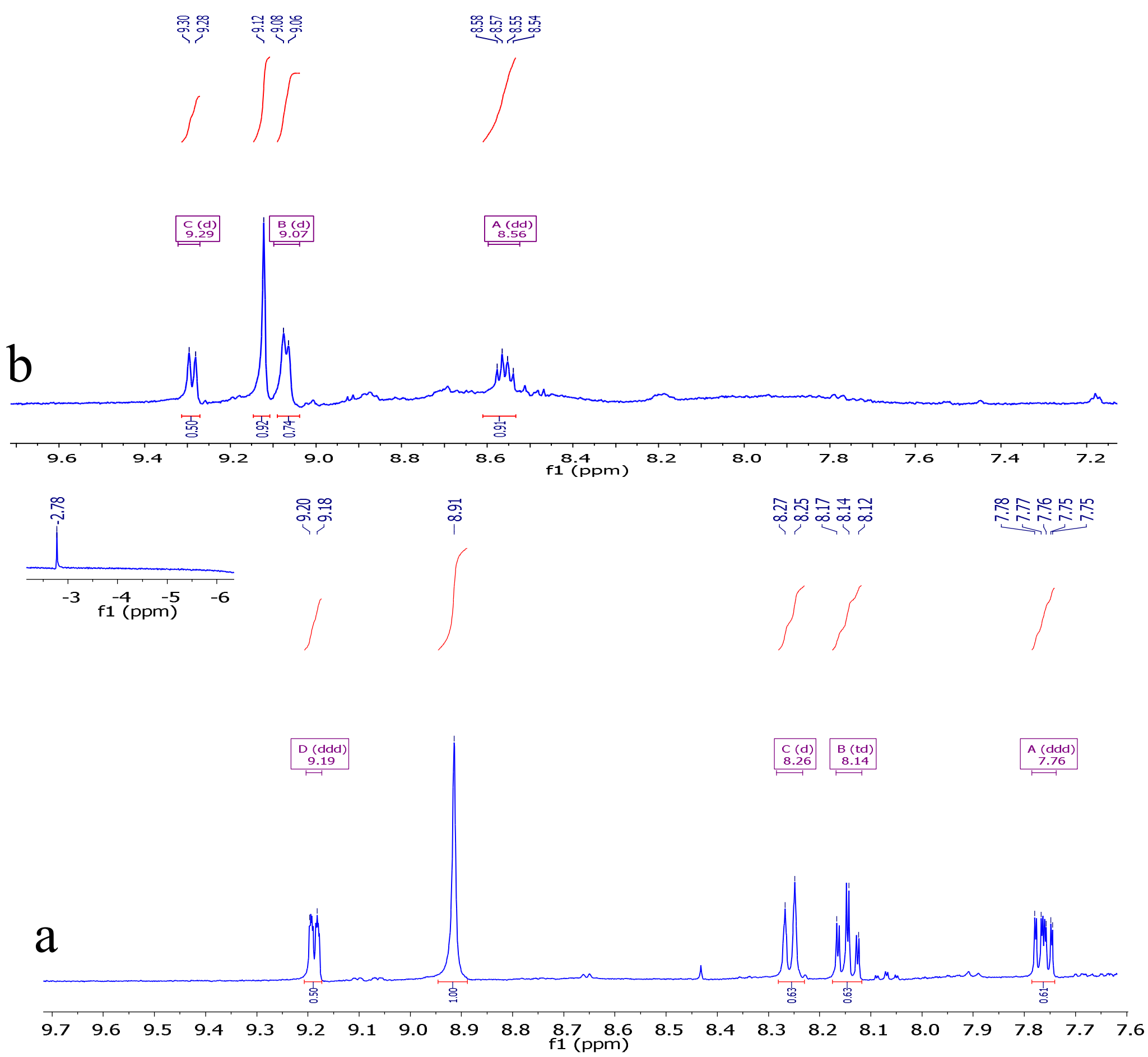

Figure S4. a) ^{1}H NMR spectra of the aromatic region of $H_2T(2\text{-}Py)P$ (in $CDCl_3$) and $H_8T(2\text{-}Py)P(Cl)_6$ (in D_2O). The inset shows the NH region of $H_2T(2\text{-}Py)P$.

S4. General oxidation procedure

Photooxidation of methyl phenyl sulfide and organic dyes (Brilliant green and Rhodamine B) was performed in a double walled glass vessel. In order to maintain a constant temperature, water was circulated through the outer jacket. Details of the reaction setup were presented elsewhere[1]. The reactions were conducted under the irradiation of a 10 W white LED lamp (see [2] for the emission spectrum of the LED lamp) (see Supplementary material S4 for more details). Aerobic photooxidation of methyl phenyl sulfide was conducted in the presence of the free base porphyrins under homogeneous conditions; in all reactions, 6.6×10^{-4} mmol of porphyrin photosensitizer (1.0 mL, 6.6×10^{-4} M in acetonitrile) and methyl phenyl sulfide (1.98 mmol) were added to 10 mL acetonitrile to achieve a 1:3000 molar ratio. In the case of the hexaprotonated derivatives 6.6×10^{-4} mmol of the photosensitizer (1.0 mL, 6.6×10^{-4} M in water) and the sulfide (1.98 mmol) were dissolved in 10 mL distilled water. The solution was saturated with oxygen by bubbling oxygen (30 min) from a cylinder through the solution. Also, the setup was equipped with an oxygen balloon connected to the glass vessel through a septum and a magnetic stir bar. The progress of reaction was followed by TLC and GC. For the reactions conducted in water, at different time intervals, an aliquot of 2 mL was taken and extracted 2 mL with 2 mL ethyl acetate. The organic phase was dried on magnesium sulfate before analysis by TLC or GC. Authentic samples of methyl phenyl sulfoxide[3] and methyl phenyl sulfone[4] prepared using the reported procedures were used to characterize the products. All reactions were triplicated and the average values were reported.

For the reactions performed under heterogeneous conditions, 0.0093 g of the polymer supported hexaprotonated porphyrin (corresponding to 6.6×10^{-4} mmol of porphyrin photosensitizer) and sulfide (1.98 mmol) were used in a 1:3000 molar ratio in 10 mL distilled water. The organic

phase prepared after the extraction of an aliquot of 2 mL of the solution, dried on magnesium sulfate was analyzed by TLC or GC.

In the case of organic dyes, the catalyst (0.0003 mmol) and substrate (0.009 mmol) were used in a 1:30 molar ratio. With the exception of the reaction performed in the presence of the free base porphyrins in acetonitrile, the other reactions were conducted in distilled water. The progress of the degradation of dye was monitored by UV–vis spectroscopy; the degradation of Brilliant green (BG) and Rhodamine B (RhB) was accompanied with the decrease in the intensity of the absorption band at 625 nm (ε = 9928.2 M^{-1} cm^{-1}) and 554 nm (ε = 104375 M^{-1} cm^{-1}), respectively. The yield of degradation of RhB and BG was determined by Beer-Lambert law in the linear part of an absorbance *vs*. concentration calibration curve constructed for each dye. The molar absorptivity of BG and RhB at the λ_{max} of the dyes (ε = 9928.2 $M^{-1}cm^{-1}$ at 625 nm, for BG and 104375.5 $M^{-1}cm^{-1}$ at 553 nm for RhB**)** determined form the calibration curves were used for the evaluation of the yields of the reactions. At different time intervals, an aliquot of 1 mL of the reaction mixture was taken and diluted to 10 mL, before recording the UV-vis spectrum to perform the measurements in the linear part of the calibration curves in which the Beer-Lambert law was obeyed. A 10 W white LED lamp with emission bands in the range of 600 to 640 nm and maximum intensity at 615 nm was used as the light source (see [2] for the emission spectrum). For the heterogeneous reactions, the catalyst was separated by centrifugation, before taking an aliquot for further dilution.[5]

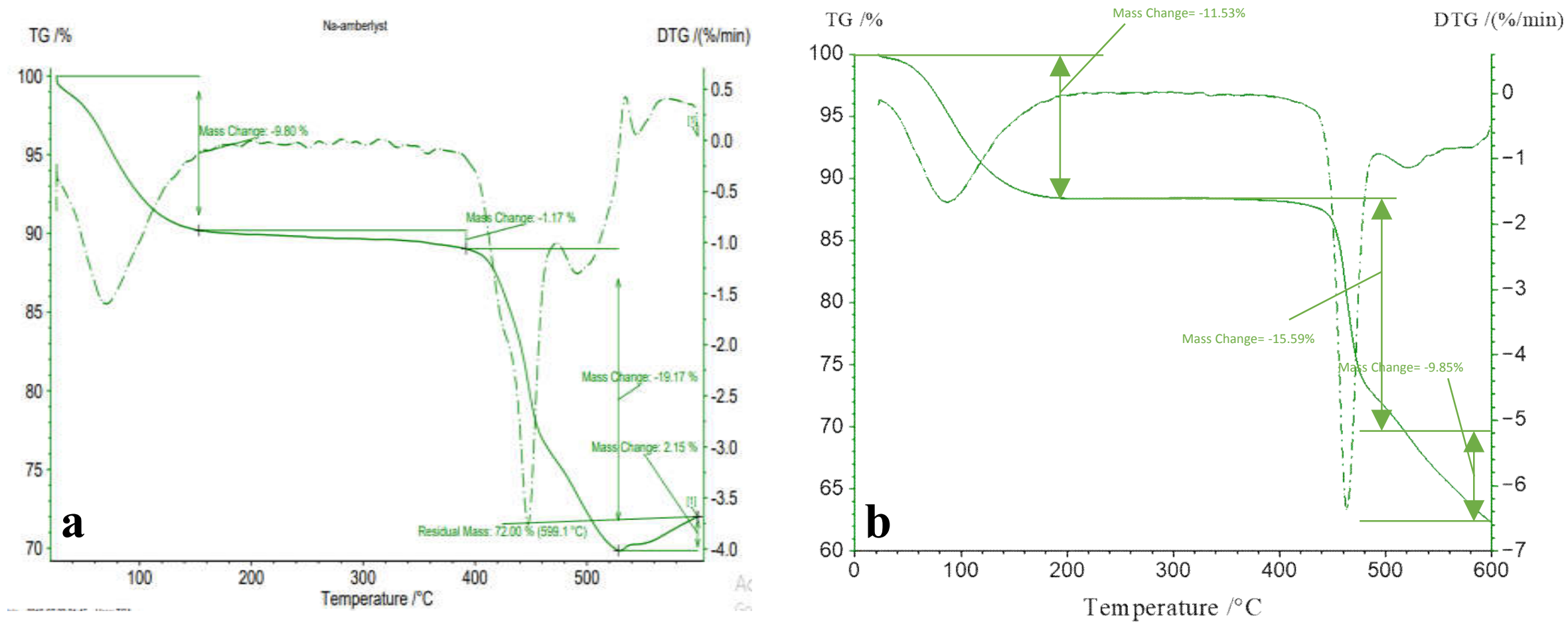

Figure S5.TGA curves of (a) nanoAmbSO_3H and (b) nanoAmbSO_3Na.

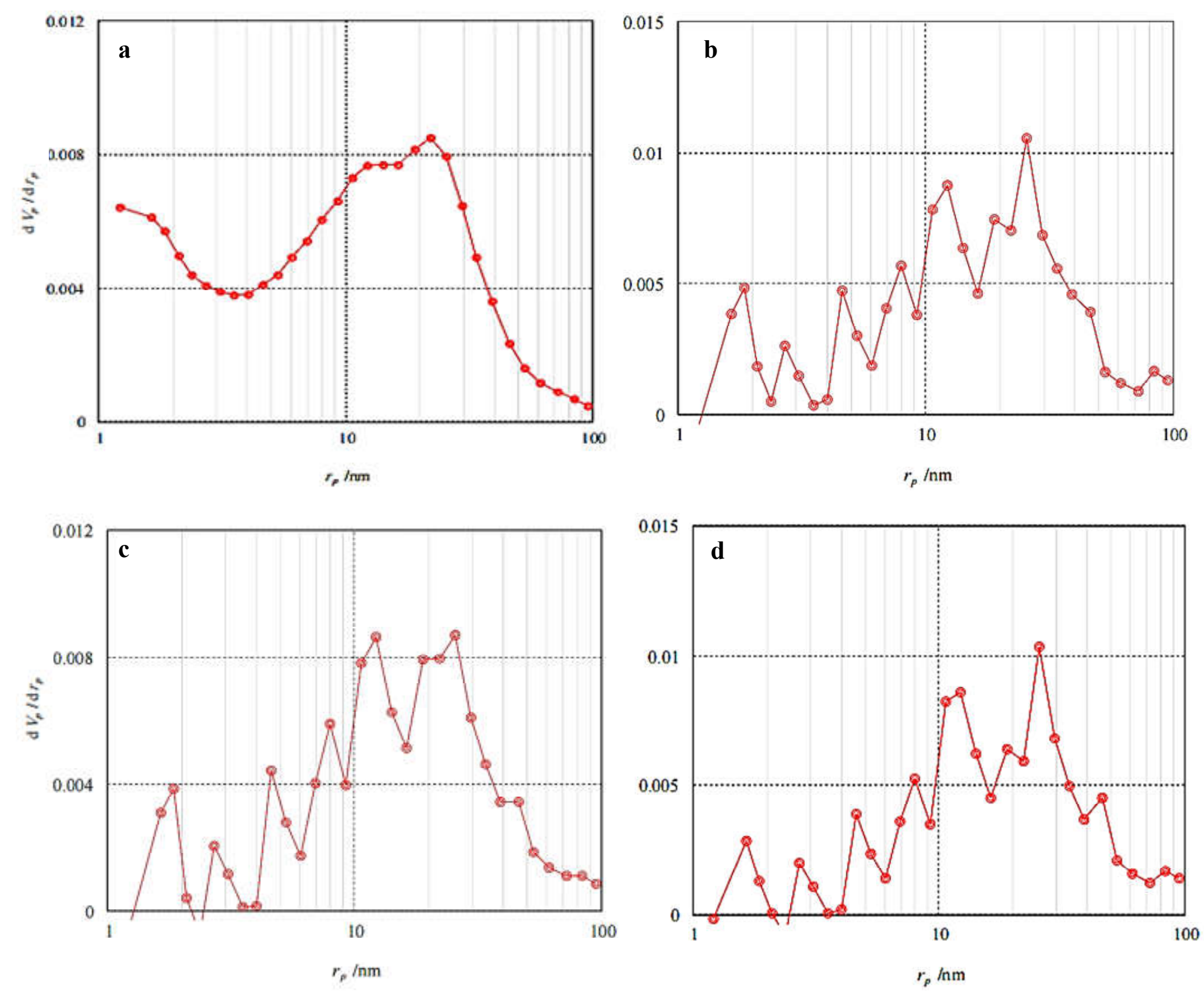

Figure S6. The BJH curves for N_2 adsorption/desorption isotherm of nanoAmbSO_3H (a), nanoAmbSO_3H@H_8T(2-Py)P (b), nanoAmbSO_3H@H_8T(3-Py)P (c), nanoAmbSO_3H@H_8T(4-Py)P (d).

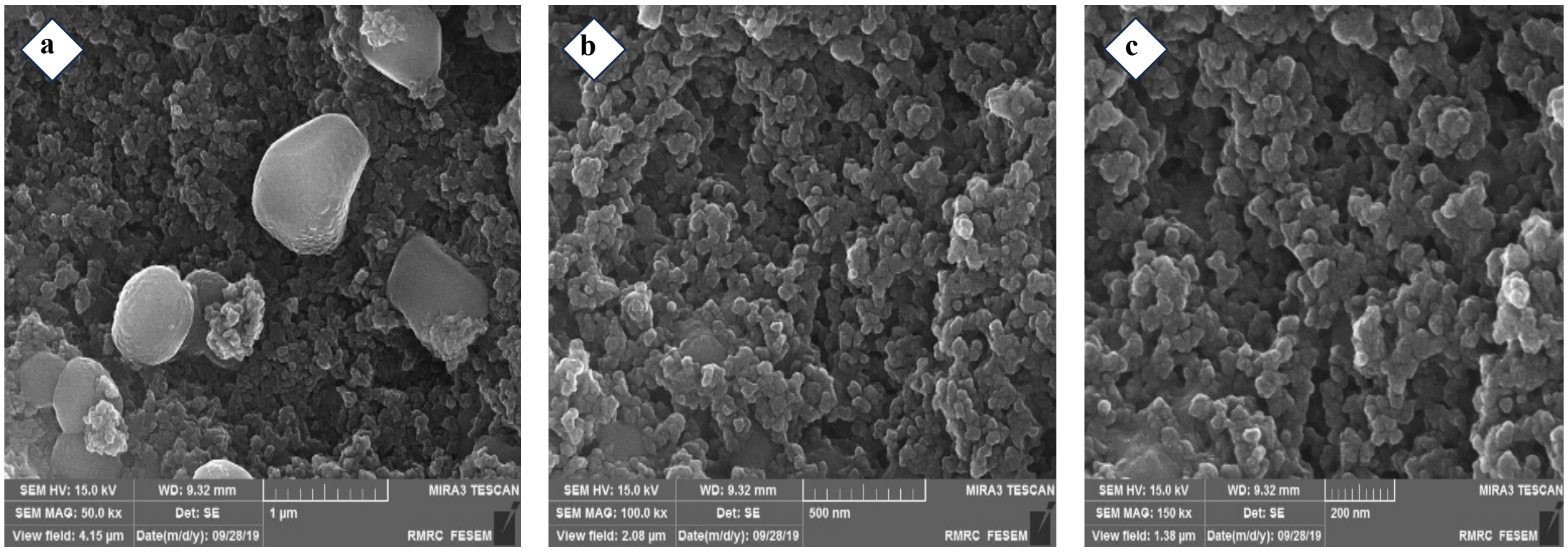

Figure S7. FESEM images of nanoAmbSO_3H magnified at a) 1µm, c) 500 nm and d) 200 nm.

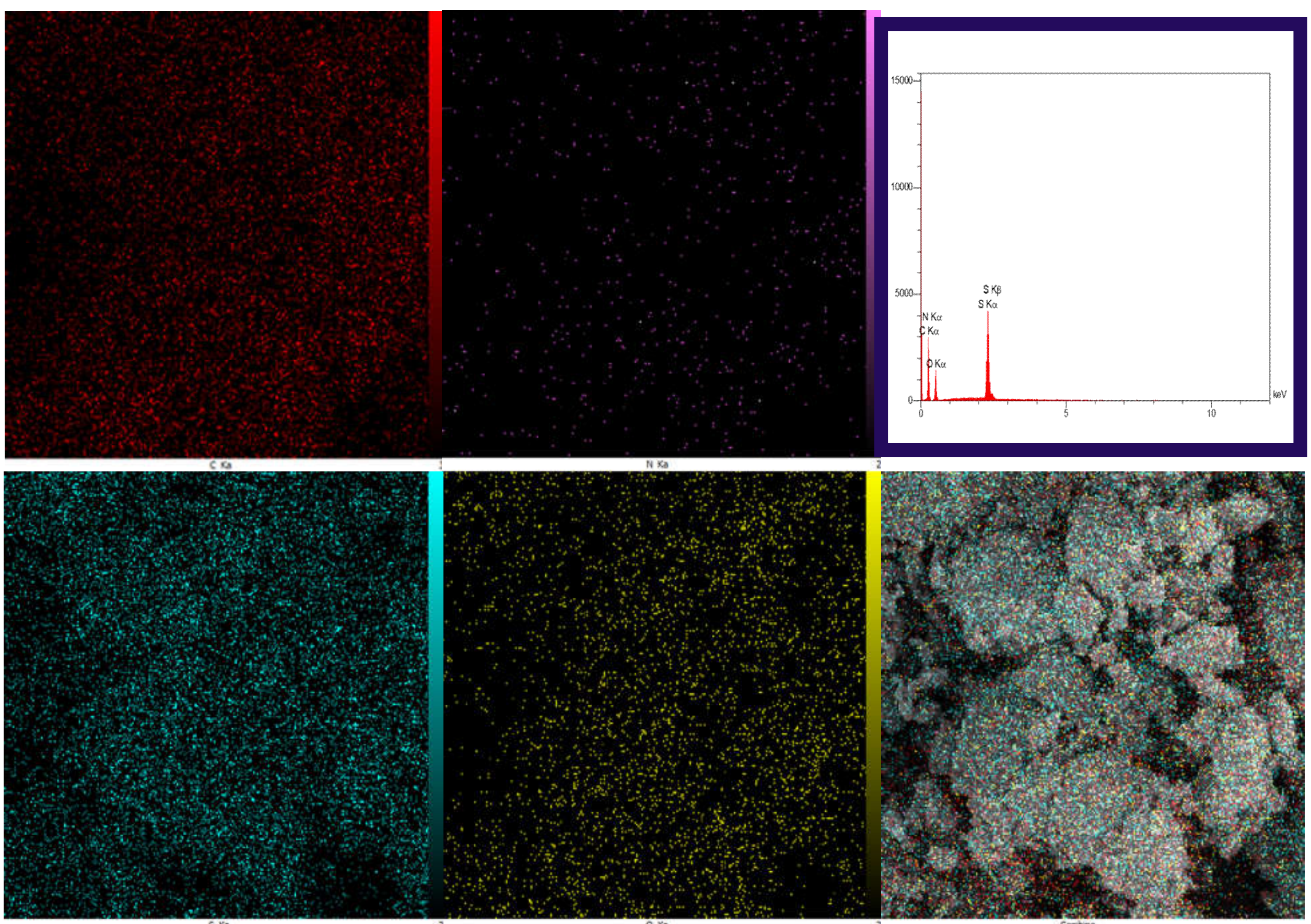

Figure S8. EDX elemental mapping of nanoAmbSO_3H@H_8T(3-Py)P.

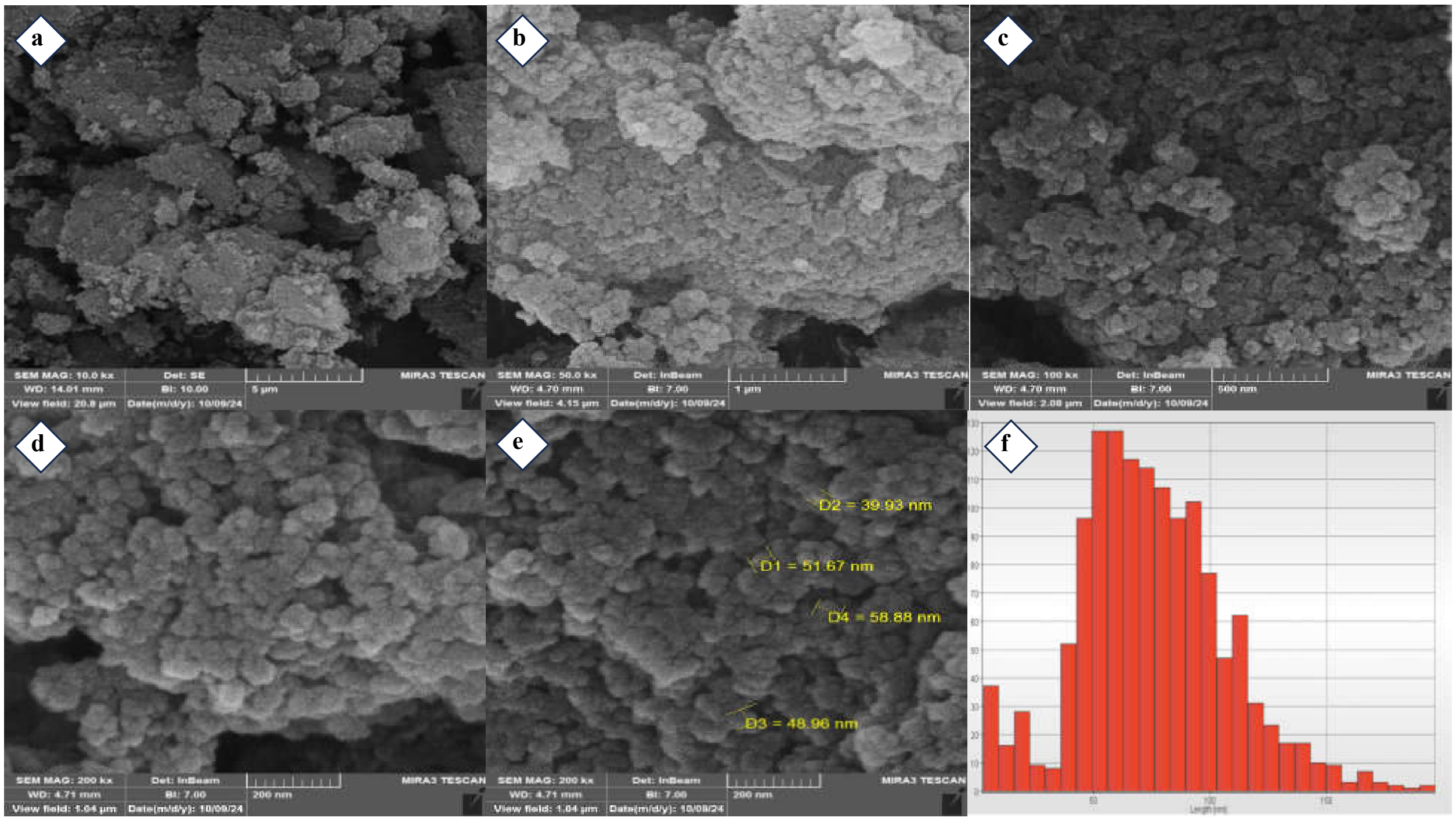

Figure S9. FESEM images of nanoAmbSO_3H@H_8T(3-Py)P magnified at a) 5 μm, b) 1 μm, c) 500 nm and d,e) 200 nm.

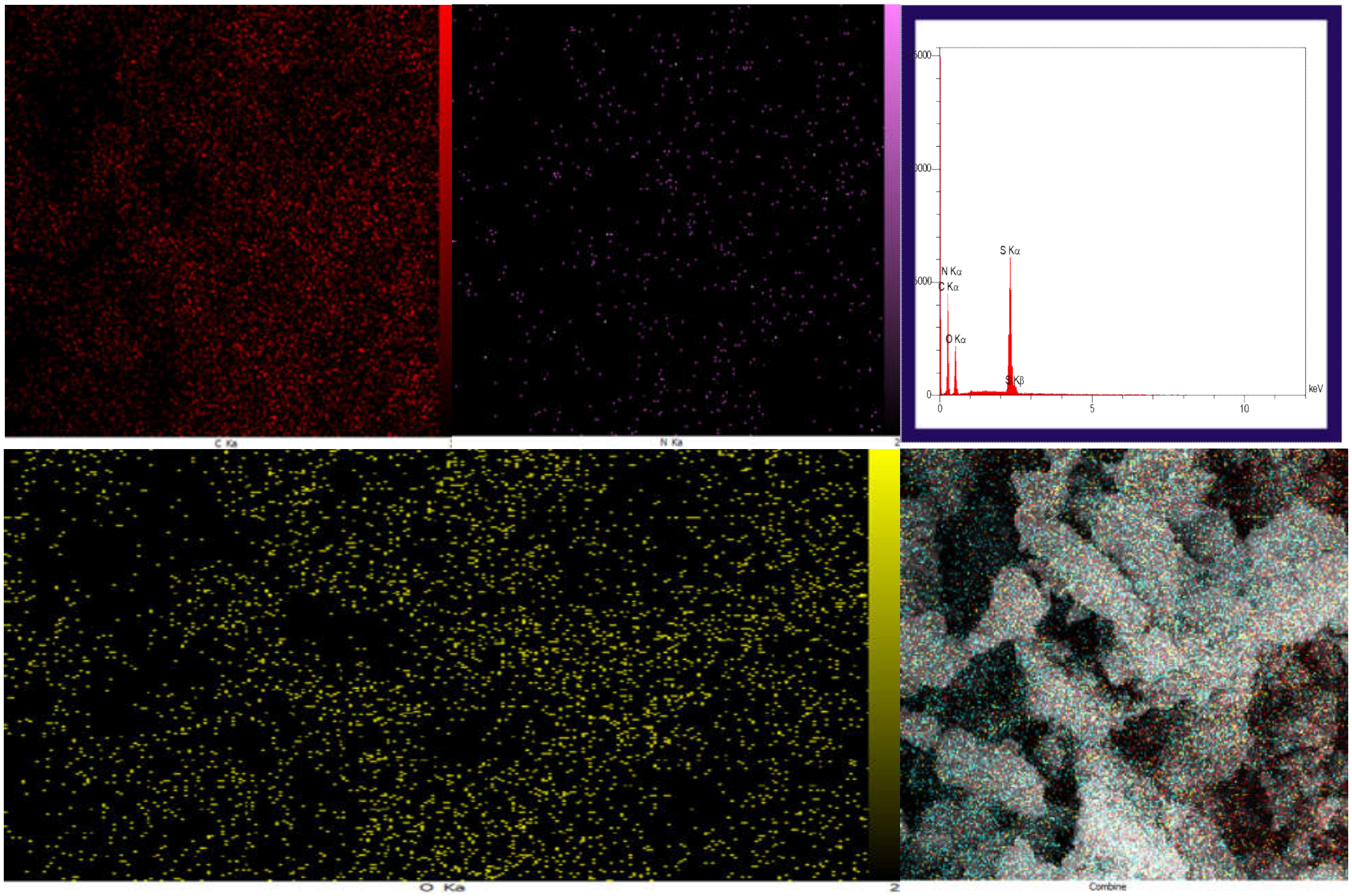

Figure S10. EDX elemental mapping of nanoAmbSO_3H@H_8T(4-Py)P.

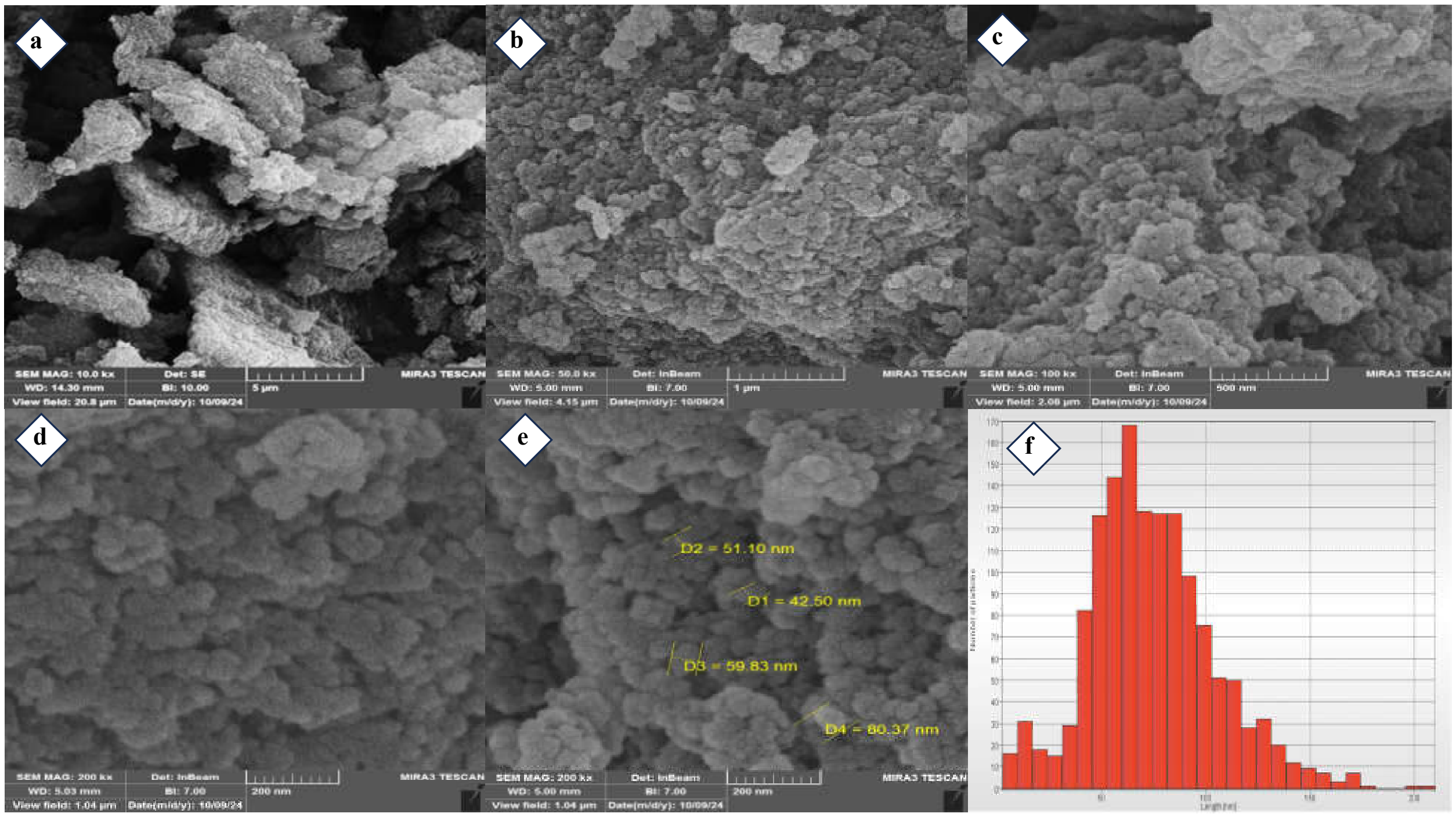

Figure S11. FESEM images of nanoAmbSO$_3$H@H$_8$T(4-Py)P magnified at a) 5 µm, b) 1 µm, c) 500 nm and d,e) 200 nm.

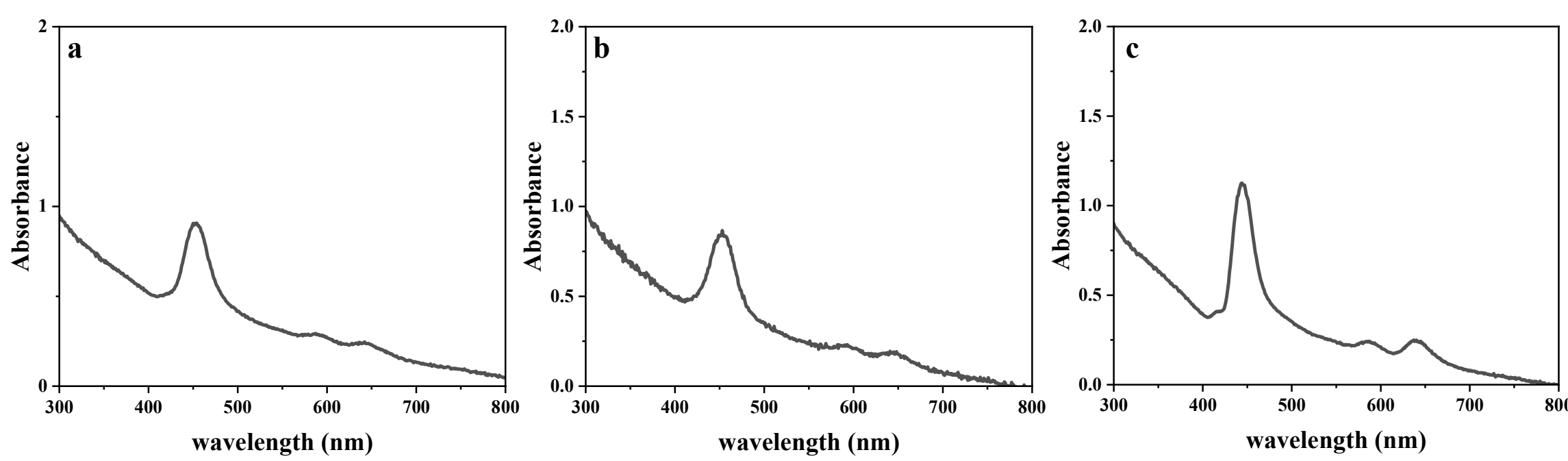

Figure S12. UV-vis spectrum of solution-like suspension of a) nanoAmbSO$_3$H@H$_8$T(2-Py)P , b) nanoAmbSO$_3$H@H$_8$T(3-Py)P , c) nanoAmbSO$_3$H@H$_8$T(4-Py)P in distilled water.

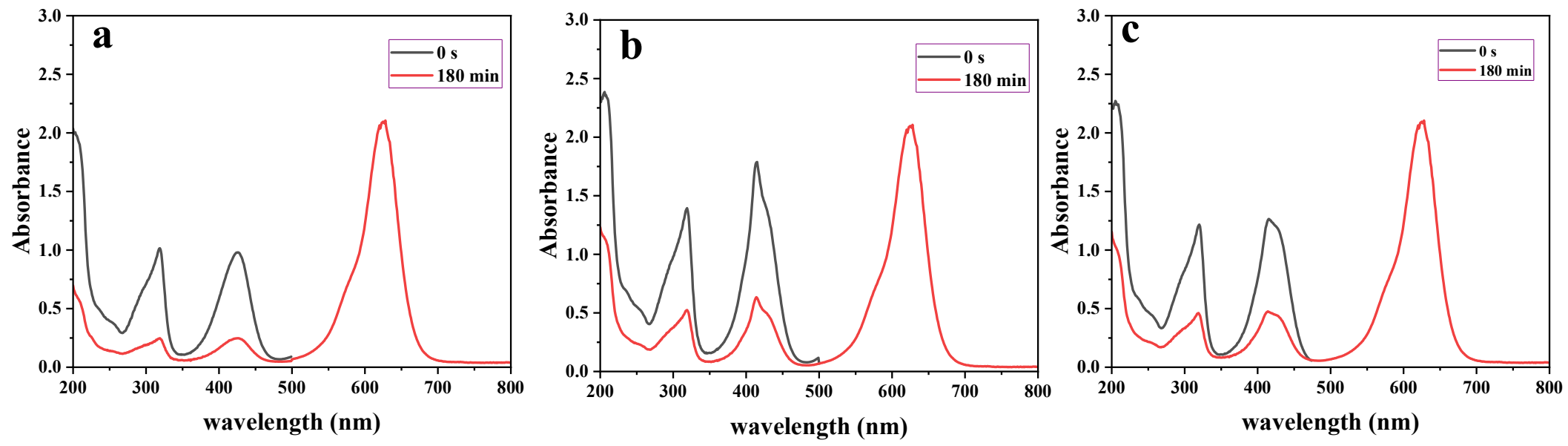

Figure S13. UV–vis spectral changes upon the aerobic oxidation of BG in the presence of a) $H_2T(2\text{-}Py)P$, b) $H_2T(3\text{-}Py)P$ and c) $H_2T(4\text{-}Py)P$.

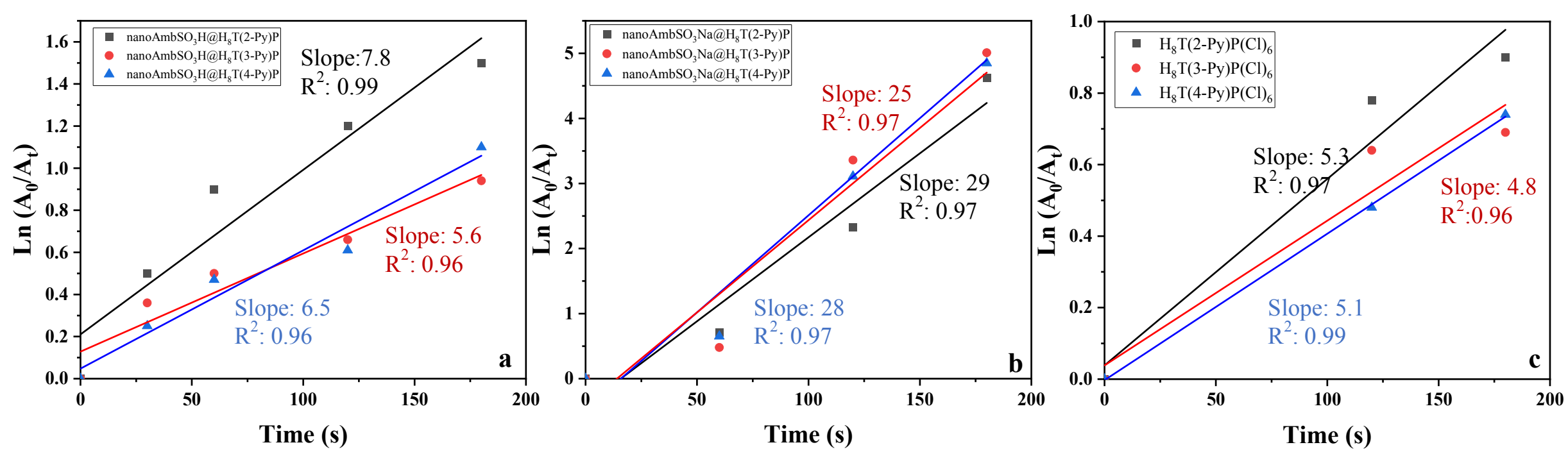

Figure S14. Kinetics curve for degradation of BG in the presence of the nanocomposites (a and b) and the water soluble porphyrin photosensitizers (c). No reaction was observed in the presence of the free base porphyrins in acetonitrile.

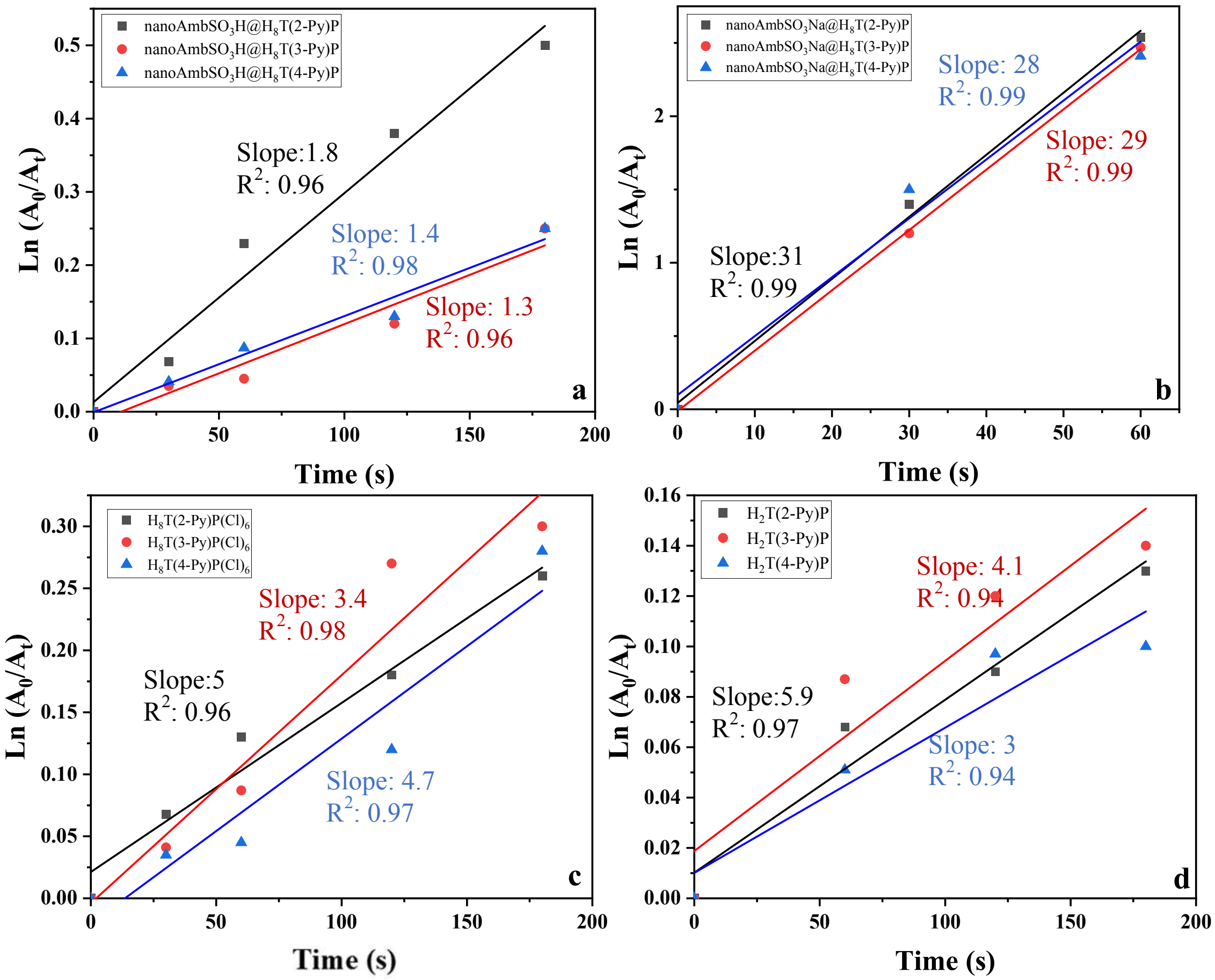

Figure S15. Kinetics curve for degradation of RhB in the presence of the nanocomposites (a and b), the water soluble porphyrin photosensitizers (c) and the free base porphyrins (in acetonitrile).

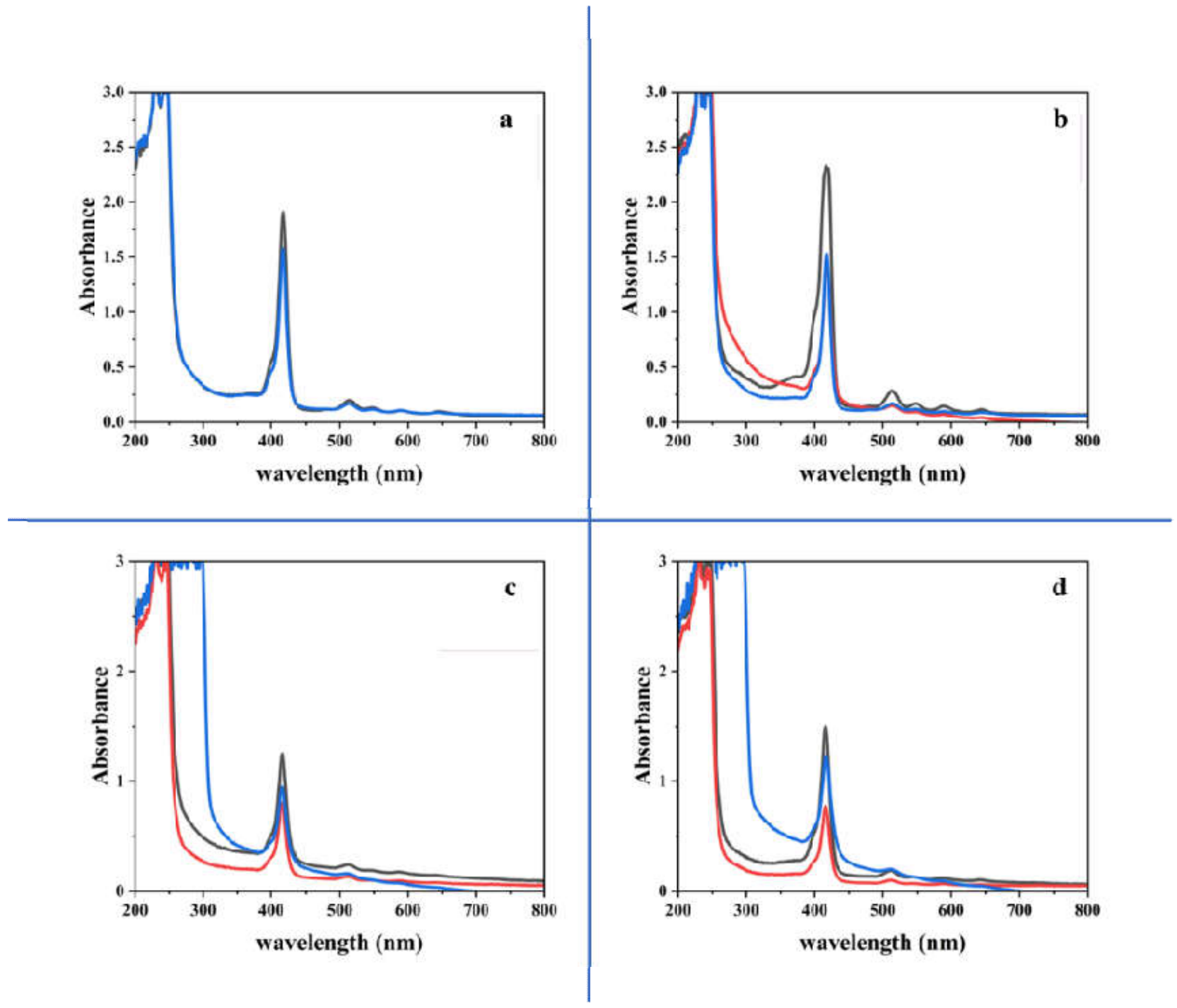

Figure S16. The oxidative degradation of the porphyrins determined both in the absence (red curve) and the presence of the substrate (blue curve), a) nanoAmbSO_3Na@H_8T(4-Py)P, b) nanoAmbSO_3H@H_8T(4-Py)P, c) nanoAmbSO_3Na@H_8T(3-Py)P and d) nanoAmbSO_3H@H_8T(3-Py)P.

References

1 A. Heydari-turkmani and S. Zakavi, *J. Catal.*, 2028, **364**, 394-405.

2 R. Nasrollahi, A. Heydari-turkmani and S. Zakavi, *Catal. Sci. Technol.*, 2029, **9**, 1260-1272.

3 M. H. Ali and G. J. Bohnert, *Synthesis*, 1998, 1238-1240.

4 N. Iranpoor, D. Mohajer and A.-R. Rezaeifard, *Tetrahedron Lett.*, 2004, **45**, 3811-3815.

5 S. R. Gamelas, J. P. Tomé, A. C. Tomé and L. M. Lourenço, *Catal. Sci. Technol.*, 2024, **14**, 2352-2389.
